# *Stenotrophomonas maltophilia* impedes *Bacillus* biocontrol of tomato wilt disease by degrading its lipopeptide antibiotics

**DOI:** 10.1093/ismejo/wraf210

**Published:** 2025-09-23

**Authors:** Junwei Peng, Dmitri V Mavrodi, Jiasui Li, Suhelen Egan, Huanhuan Zhang, Xiuli Fan, Yang Liu, Keke Dang, Olga V Mavrodi, Qin Liu, Yuanhua Dong, Jiangang Li

**Affiliations:** State Key Laboratory of Soil and Sustainable Agriculture, Institute of Soil Science, Chinese Academy of Sciences, Nanjing, Jiangsu 211135, China; University of Chinese Academy of Sciences, Beijing 100049, China; University of Chinese Academy of Sciences, Nanjing, Jiangsu 211135, China; School of Biological, Environmental, and Earth Sciences, The University of Southern Mississippi, Hattiesburg, MS 39406, United States; Centre for Marine Science and Innovation, School of Biological, Earth and Environmental Sciences, The University of New South Wales, Sydney, NSW 2052, Australia; Centre for Marine Science and Innovation, School of Biological, Earth and Environmental Sciences, The University of New South Wales, Sydney, NSW 2052, Australia; State Key Laboratory of Soil and Sustainable Agriculture, Institute of Soil Science, Chinese Academy of Sciences, Nanjing, Jiangsu 211135, China; University of Chinese Academy of Sciences, Beijing 100049, China; University of Chinese Academy of Sciences, Nanjing, Jiangsu 211135, China; State Key Laboratory of Soil and Sustainable Agriculture, Institute of Soil Science, Chinese Academy of Sciences, Nanjing, Jiangsu 211135, China; University of Chinese Academy of Sciences, Beijing 100049, China; University of Chinese Academy of Sciences, Nanjing, Jiangsu 211135, China; State Key Laboratory of Soil and Sustainable Agriculture, Institute of Soil Science, Chinese Academy of Sciences, Nanjing, Jiangsu 211135, China; University of Chinese Academy of Sciences, Beijing 100049, China; University of Chinese Academy of Sciences, Nanjing, Jiangsu 211135, China; State Key Laboratory of Soil and Sustainable Agriculture, Institute of Soil Science, Chinese Academy of Sciences, Nanjing, Jiangsu 211135, China; School of Biological, Environmental, and Earth Sciences, The University of Southern Mississippi, Hattiesburg, MS 39406, United States; State Key Laboratory of Soil and Sustainable Agriculture, Institute of Soil Science, Chinese Academy of Sciences, Nanjing, Jiangsu 211135, China; University of Chinese Academy of Sciences, Nanjing, Jiangsu 211135, China; State Key Laboratory of Soil and Sustainable Agriculture, Institute of Soil Science, Chinese Academy of Sciences, Nanjing, Jiangsu 211135, China; University of Chinese Academy of Sciences, Nanjing, Jiangsu 211135, China; State Key Laboratory of Soil and Sustainable Agriculture, Institute of Soil Science, Chinese Academy of Sciences, Nanjing, Jiangsu 211135, China; College of Agricultural Science and Engineering, Hohai University, Nanjing, Jiangsu 211100, China; College of Soil and Water Conservation, Hohai University, Nanjing, Jiangsu 211100, China; University of Chinese Academy of Sciences, Beijing 100049, China; University of Chinese Academy of Sciences, Nanjing, Jiangsu 211135, China

**Keywords:** Ralstonia solanacearum, Stenotrophomonas maltophilia, Bacillus, bacterial interaction, antibiotic inactivation, lipopeptides, biocontrol

## Abstract

Harnessing antibiotic-producing microorganisms that antagonize pathogens represents a sustainable approach for plant disease management. However, biocontrol agents that are effective in the laboratory often have diminished or variable performance in the field. It is often assumed that microbial interactions within the plant rhizosphere can influence the performance of biocontrol agents. To validate this hypothesis, we established a tripartite bacterial model system based on field investigations, involving antibiotic producers (*Bacillus amyloliquefaciens* P224, *Bacillus subtilis* P165, and *Bacillus velezensis* P63), an antibiotic degrader (*Stenotrophomonas maltophilia* P373), and a bacterial plant pathogen (*Ralstonia solanacearum* PA1)*.* The selected *Bacillus* species antagonize *R. solanacearum* and act as biocontrol agents of the bacterial wilt of tomatoes caused by this pathogen. We demonstrated that *S. maltophilia* diminished this biocontrol effect by degrading the lipopeptide antibiotics iturin, fengycin, and surfactin secreted by *Bacillus* spp., thereby serving as a “pathogen helper” that indirectly facilitated pathogen invasion. Further transcriptomic and proteomic analyses revealed that the lipopeptide inactivation mechanism in *S. maltophilia* involved multidrug efflux systems, ribosomal adaptation, and enzymatic hydrolysis. Additionally, the interspecies interactions in our model system are modulated by nutrient availability, with elevated carbon sources enhancing the interference competitive ability of *Bacillus* spp. against *S. maltophilia*, thereby mitigating its negative impact on the biocontrol of *R. solanacearum*. Our study sheds light on the complex interactions among plant pathogens, biocontrol agents, and the indigenous microbial community, underscoring the necessity to account for native antibiotic-degrading organisms when applying biocontrol strategies for effective disease management.

## Introduction

Plant diseases significantly threaten global crop production, food security, and ecosystem health [1, 2]. *Ralstonia solanacearum* is a ubiquitous, economically important pathogen of over 200 plant species and a causative agent of wilts, brown rot, and Moko disease that devastate crop production [3, 4]. Among particularly destructive *R. solanacearum* diseases is the bacterial wilt of tomato (*Solanum lycopersicum*) that, under favorable conditions, can result in up to 90% yield reductions and substantial economic losses [5]. Despite the implementation of crop rotations, various soil treatments, and grafting of popular fruiting varieties onto resistant rootstocks [6, 7], effective and environmentally sustainable control strategies for the bacterial wilt of tomatoes remain elusive. Managing microbiota has the potential to enhance plant fertility and yield while suppressing key pathogens*,* and there is growing interest in biological control to mitigate this disease [8, 9]. As typical and frequently dominant members of plant-associated microbial communities, bacterial genera such as *Bacillus*, *Pseudomonas*, and *Streptomyces* are potent producers of numerous antibacterial and antifungal compounds [10–12], and they have been formulated as microbial pesticides, fungicides, and fertilizers, with commercial products such as *Bacillus subtilis*, *Pseudomonas chlororaphis*, and *Streptomyces lydicus* [13]. However, despite years of active studies, such strains often show limited effectiveness in the field [14, 15] due to significant gaps in our understanding of the complex rhizosphere processes. The plant rhizosphere—the narrow soil region shaped by root exudates and soil microbial activity—is a biologically rich environment where complex microbial networks orchestrate plant–microbe interactions that critically influence plant health [16, 17]. Indigenous microflora can affect the establishment and effectiveness of antibiotic-producing biocontrol agents through direct mechanisms including nutrient supply [18], biofilm formation [19], and niche competition [20], as well as indirectly, by altering plant–microbe interactions through changes in root exudate profiles [21] and host immune responses [22].

Recent studies of interspecies interactions have reported that the effectiveness of antibiotics against target species can be diminished by the presence of “disruptor” strains [23, 24]. Typically, such disruptors exhibit high antibiotic resistance and possess the ability to inactivate antibiotics [25, 26]. Consequently, the presence of antibiotic-degrading species can alter the effectiveness of antibiotics against antibiotic-sensitive community members [27]. For example, a detailed clinical antibiotic therapy study demonstrated that *Stenotrophomonas maltophilia* can hydrolyze and detoxify imipenem by secreting β-lactamases, thereby providing protection to imipenem-sensitive *Pseudomonas aeruginosa* [28]. Emerging evidence has revealed analogous antibiotic inactivation mechanisms in agricultural biocontrol agents, where cyclic lipopeptides (CLPs) antibiotics produced by beneficial *Bacillus* and *Pseudomonas* strains undergo enzymatic degradation by specific microbiota, such as *Streptomyces venezuelae* [29], *Stenotrophomonas indicatrix* [30], and *Mycetocola* spp. [31]. We posit that such microbial detoxification operates in the plant rhizosphere and that antibiotic-degrading microorganisms can inactive antimicrobials produced by biocontrol agents, thereby acting as “pathogen helpers” and facilitating the pathogen infection of host plants. We also hypothesize that the interactions between antibiotic producers, degraders, and pathogens are modulated by environmental factors.

**Figure 1:**
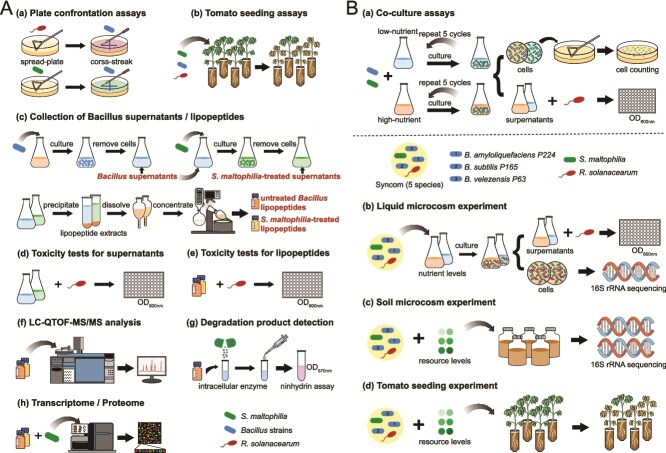
Experimental procedures. (A) Characterization of biological mechanisms underlying interactions between *S. maltophilia* and LP-producing *Bacillus* spp. (a) Plate confrontation assays to evaluate the antagonistic effects of *Bacillus* strains on *R. solanacearum* PA1 and *S. maltophilia* P373; (b) tomato seedling assays to assess the impact of *S. maltophilia* on *Bacillus*-mediated control of tomato bacterial wilt; (c) preparation and processing of *S. maltophilia*-treated and -untreated *Bacillus* culture supernatants for LP extraction; toxicity evaluation of *Bacillus* supernatants (d) and LPs (e) against the pathogen *R. solanacearum*; (f) identification of *Bacillus* LPs using ESI-TOF-MS; (g) assessment of LP degradation by the ninhydrin method; (h) transcriptomic and proteomic analysis of *S. maltophilia* in responses to *Bacillus* LPs; (B) the influence of resource availability on the interactions among *Bacillus* spp., *S. maltophilia*, and *R. solanacearum*. (a) Co-culture competitive assays between *Bacillus* and *S. maltophilia* under low and high nutrient availability; validation of the nutrient availability effect on interspecies interactions using liquid co-cultures (b), soil microcosms (c), and plant assays (d).

To test our hypotheses, we focused on the bacterial wilt of tomatoes and utilized a tripartite model system involving an antibiotic-producing biocontrol agent, an antibiotic degrader, and the plant pathogen *R. solanacearum* (Fig. 1). In this system, the antibiotic producers were represented by biocontrol strains of *B. amyloliquefaciens*, *B. subtilis*, and *B. velezensis*, which nonribosomally synthesized cyclic lipopeptides (LPs) active against the pathogen *R. solanacearum* [32, 33]. The antibiotic degrader *S. maltophilia* was chosen for its ubiquitous presence in rhizosphere microbial communities and resistance to a wide range of antibiotics [34, 35]. Our goal was to determine how bacterial interactions within this tripartite system impact disease progression. We further aimed to characterize the effect of environmental factors since it is known that the production of antibiotics by biocontrol strains is modulated by temperature, water activity, and pH [36–38]. Finally, we explored how interactions within our model system are affected by the nutrient availability influencing the growth and metabolic activity of bacteria. In the rhizosphere, these nutrients are supplied in the form of root exudates that shape the composition of microbial communities and interspecies interactions [39, 40].

## Materials and methods

### Field experiment and sampling of rhizosphere soil

Rhizosphere soil samples from diseased and healthy plants were collected from tomato plants cultivated in a high tunnel located near the town of Hengxi (31° 73′ N and 118° 78′ E), Nanjing, Jiangsu province of China, during the harvest period (July 2021). In this greenhouse, the tomatoes have been grown under long-term monoculture since 2016. Ten diseased [41] plants showing severe wilt symptoms (75%–100% of leaves wilted or dead) and 10 healthy plants adjacent to the diseased ones were selected for further analyses (Supplementary Fig. S1A). Rhizosphere soil samples from the diseased and healthy plants were collected, passed through a 2-mm sieve to remove plant tissue and debris, and placed at −80°C for subsequent pathogen quantification by qPCR and 16S-based microbiome profiling.

### Strains and media

The pathogen *R. solanacearum* PA1, the antibiotic degrader *S. maltophilia* P373, and antibiotic-producing biocontrol strains *B. amyloliquefaciens* P224, *B. subtilis* P165, and *B. velezensis* P63 were all isolated from the rhizosphere of tomato (*Lycopersicon esculentum*, cultivar Hezuo 903) exhibiting symptoms of bacterial wilt. The strains were taxonomically classified based on 16S rRNA gene and whole-genome sequencing, with the data provided in the NCBI BioSample and GenBank database (accession number PRJNA1296821, PRJNA1295747, PRJNA1295742, PRJNA1295741, and PP471246).

Strains were routinely cultivated in base growth (BG) media containing 3 g l^−1^ yeast extract, 3 g l^−1^ tryptone, 5 g l^−1^ glucose, 10 mM K_2_HPO_4_, 5.5 mM KH_2_PO_4_, 1 mM MgSO_4_, and 0.1 mM CaCl_2_, pH adjusted to 7.0. Bacteria were enumerated by spread plating on the Luria-Bertani (LB) medium containing 5 g l^−1^ yeast extract, 10 g l^−1^ tryptone, 10 g l^−1^ NaCl, and 17 g l^−1^ agar.

### Plate confrontation assays evaluating pairwise interactions among *R. solanacearum*, *S. maltophilia*, and *Bacillus* spp.

The antagonistic effect of *Bacillus* strains (*B. amyloliquefaciens* P224, *B. subtilis* P165, and *B. velezensis* P63) against *S. maltophilia* p373 and *R. solanacearum* PA1 was evaluated using confrontation assays on Casamino acid–peptone–glucose (CPG) agar (10 g l^−1^ peptone, 5 g l^−1^ glucose, 1 g l^−1^ acid hydrolyzed casein, 17 g l^−1^ agar, pH 7.0) (Fig. 1A, a). Briefly, CPG plates were spread-inoculated with 100 μl of either PA1 or P373 suspension (OD_600_ = 0.2), followed by cross-streaking each *Bacillus* strain at the center of the plate. The growth of PA1 and P373 was assessed after 2 days of incubation at 30°C. The same protocol was used to evaluate *S. maltophilia* P373 against *Bacillus* strains and *R. solanacearum* PA1. Briefly, CPG plates were spread-inoculated with either *Bacillus* or PA1 suspensions (OD_600_ = 0.2), followed by cross-streaking *S. maltophilia* P373.

### Toxicity assessment of *Bacillus* culture supernatants treated with *S. maltophilia*

To evaluate whether *S. maltophilia* P373 inactivates antibacterial metabolites of *B. amyloliquefaciens* P224, *B. subtilis* P165, and *B. velezensis* P63, we performed toxicity assessments of *Bacillus* culture supernatants following exposure to *S. maltophilia* (Fig. 1A, c). Briefly, 10 μl of the overnight culture of each *Bacillus* strain (OD_600_ = 1.0) were inoculated into 20 ml of BG medium. After 30 h of incubation at 30°C and 150 rpm, the bacterial cells were separated by centrifugation for 5 min at 10 000 × g and the clarified *Bacillus* spent media were collected and sterilized by filtration through a sterile 0.22 μm filter. For *S. maltophilia*-treated supernatants, 20 ml of the *Bacillus* spent media were inoculated with 100 μl of *S. maltophilia* P373 cell suspension (OD_600_ = 2.0). After an incubation at 30°C with shaking (150 rpm) for 30 h, the *S. maltophilia*-treated supernatants were clarified by centrifugation and filtration through the 0.22 μm filter. Both *S. maltophilia*-treated and -untreated *Bacillus* supernatants were stored at −20°C for subsequent toxicity assays and LP extraction.

The antimicrobial toxicity was assessed by measuring the growth of *R. solanacearum* PA1 in the presence of untreated and *S. maltophilia*-treated supernatants (Fig. 1A, d). Briefly, 5-μl aliquots of overnight pathogen culture (OD_600_ = 0.1) were mixed in a 96-well plate with 20, 50, 100, and 200 μl of sterilized *Bacillus* supernatants or *S. maltophilia*-treated supernatants. The total volume in each well was adjusted to 200 μl with fresh BG medium. Each treatment had four replicates. Uninoculated supernatant controls (200 μl supernatant alone) were included to verify the absence of residual cells. The plates were incubated at 30°C with shaking (150 rpm). After 24 h, the growth of pathogen *R. solanacearum* PA1 was quantified by measuring the OD_600_ absorbance of cultures with a SpectraMax M5 microplate reader. The complete absence of detectable growth (OD_600_ < 0.05) in uninoculated control wells confirmed effective cell removal and validated the experimental results.

### Bioassay-guided isolation and toxicity assessment of *S. maltophilia*-treated lipopeptide antibiotics

The lipopeptide antibiotics of *B. amyloliquefaciens* P224, *B. subtilis* P165, and *B. velezensis* P63 were obtained using established protocols [42, 43] (Fig. 1A, c). Both control *Bacillus* culture supernatants and *S. maltophilia*-treated supernatants were precipitated by adjusting pH to 2.0 with 6 M HCl followed by overnight incubation at 4°C. The precipitates were collected by centrifugation at 10 000 × g for 10 min, dissolved in methanol, and then dried in a rotary vacuum evaporator. The resultant samples were concentrated 20-fold by dissolving in methanol and used in growth inhibition assays and for instrumental analysis. For LP toxicity assessment, control extracts were serially diluted in 200 μl of BG medium at 1/64×, 1/32×, 1/16×, 1/8×, 1/4×, 1/2×, 1×, and 2× of their original concentration in the culture supernatant (corresponding to LP content of 3.125, 6.25, 12.5, 25, 50, 100, 200, and 400 μl supernatant equivalents) and inoculated with 5 μl of an overnight culture (OD_600_ = 0.2) of *R. solanacearum* PA1 or *S. maltophilia* P373.

To evaluate *S. maltophilia*-mediated detoxification (Fig. 1A, e), both control and treated extracts were serially diluted in 200 μl of BG medium at 1/10×, 1/4×, 1/2×, and 1× of their original concentration in the culture supernatant (equivalent to LP content of 20, 50, 100, and 200 μl supernatants) and challenged with 5 μl of *R. solanacearum* PA1 suspension (OD_600_ = 0.2). The cultures were incubated for 24 h at 30°C with shaking, after which the growth was evaluated by measuring absorbance at 600 nm.

### Structural characterization of lipopeptide antibiotics by LC-QTOF-MS/MS

All LP antibiotic extracts were characterized using electrospray ionization time-of-flight mass spectrometry (ESI-TOF-MS) (Fig. 1A, f). The samples were profiled using an AB SCIEX X500R LC-QTOF-MS/MS system (AB SCIEX, Framingham, MA) equipped with an ACQUITY UPLC BEH C18 column (100 mm × 2.1 mm, 1.8 μm; Waters, USA). The mobile phases consisted of (A) water containing 0.1% formic acid and 2 mM ammonium acetate and (B) acetonitrile (ACN). Analytes were separated using the following gradient program: 0–5.0 min, 75%–55% A (25%–45% B); 5.1–50.0 min, 55% A (45% B); 50.1–55.0 min, 0% A (100% B); and 55.1–60.0 min, 0%–75% A (100%–25% B). The flow rate was 0.25 ml min^−1^, and 5-μl sample aliquots were injected using an autosampler maintained at 15°C. The column temperature was kept at 40°C, and the LP profiles were scanned over a mass range of *m*/*z* 100–2000 and analysed in positive ionization modes. The generated TOF MS and TOF MS/MS data were processed using SCIEX OS software (Version 3.0). Candidate LP formulas were screened using the “Formula Finder” tool with a mass error tolerance <5.0 ppm against the ChemSpider database. For structural characterization, TOF-MS/MS analysis was performed to analyse fragment ion patterns and determine amino acid sequences of LPs.

### Ninhydrin-based detection of amino groups in *S. maltophilia*-mediated *Bacillus* lipopeptide degradation

To investigate the degradation of *Bacillus* LPs by *S. maltophilia* P373, the LP extracts were treated with intracellular enzymes of P373 (Fig. 1A, g). The degradation results in the release of amino acids, which can be quantified using a ninhydrin assay [44]. To induce the synthesis of degradative enzymes, 200 μl of LP extracts from *B. amyloliquefaciens* P224, *B. subtilis* P165, or *B. velezensis* P63 were added to a 20-ml culture of *S. maltophilia* P373 in BG medium. The control received an identical volume of methanol, and each treatment was performed in triplicate. After overnight incubation, the LP-induced and noninduced P373 cells were harvested and suspended in PBS buffer (pH = 7.0) to an OD_600_ of 1.0. Equivalent volumes of cell suspension from each treatment were lysed via intermittent sonication in an Ultrasonic Cell Crusher (XO-900D, Nanjing) for 5 min, followed by centrifugation at 10 000 × g for 5 min. The resultant cell extracts were collected and used to treat the *Bacillus* LPs. Briefly, 9 ml of each cell extract were mixed with 0, 100, or 1000 μl of an LP extract and 1000, 900, or 0 μl, respectively, of methanol. The samples were incubated at 30°C with shaking at 150 rpm for 48 h, after which 4 ml of each sample were mixed with 1 ml of ninhydrin solution (2%, w/w) and 1 ml of PBS solution (pH = 8.0). The samples were incubated for 20 min at 90°C and allowed to cool down to room temperature before quantifying amino acids by measuring the absorbance at 570 nm using a SpectraMax M5 microplate plate reader.

### Transcriptome responses of *S. maltophilia* to *Bacillus* lipopeptides

To investigate the gene responses of *S. maltophilia* P373 to LPs (Fig. 1A, h), 200 μl of P373 cells (OD_600_ = 2.0) were inoculated in 20 ml BG media supplemented with 500 μl of LP extracts from each of the studied *Bacillus* strains or methanol solvent as a control. After 36 h of incubation with shaking at 30°C, *S. maltophilia* cells were harvested by centrifugation, frozen in liquid nitrogen, and stored at −80°C for RNA extractions. Total RNA was extracted with TRIzol Reagent (Invitrogen, Beijing, China) following the manufacturer’s instructions. The quality and quantity of RNA were measured using a NanoDrop 2000 ultramicro-spectrophotometer (Thermo Scientific, Waltham, USA). The samples were treated using a cDNA reverse transcription kit (Takara Bio, Japan) and used to prepare libraries with an Illumina TruSeq RNA Sample Preparation Kit (San Diego, CA). Sequencing was performed on a NovaSeq 6000 System (Illumina; Majorbio, Shanghai, China) at 150-bp paired read mode. The resultant high-quality reads (Q30 nucleobase percentage >93.99%) were individually aligned to the reference *S. maltophilia* K279a (GenBank accession number GCA_000072485.1), resulting in matching rates ranging from 76.08% to 87.64% (Supplementary Table S1). Following the normalization of read count data by Transcripts per Million, differentially expressed genes (DEGs) were identified using the DESeq2 (Version 1.30.1), employing a filtering threshold of |log_2_ fold change| ≥ 1.0 and BH-adjusted *P* < .05. The pathway tool was then used for enrichment analysis according to the Kyoto Encyclopedia of Genes and Genomes (KEGG) pathways [45] and Gene Ontology (GO) terms [46].

### Proteomic analysis of *S. maltophilia* in response to *Bacillus* lipopeptides

To investigate the proteomic responses of *S. maltophilia* P373 to *Bacillus* LPs, the same cell cultures used for transcriptomic analysis were employed for protein extraction and analysis (Fig. 1A, h). Briefly, total protein extraction was conducted by suspending frozen cell samples in a protein lysis buffer [8 M urea, 1% sodium dodecyl sulfate (SDS)] supplemented with protease inhibitors. The samples were then homogenized using a tissue grinder, intermittently vortexed, and centrifuged at 14 000 × g for 10 min at 4°C. Protein concentration was determined by Bicinchoninic acid (BCA) method by BCA Protein Assay Kit (Thermo Scientific), and protein quality was assessed by SDS-polyacrylamide gel electrophoresis. For protein digestion, 100 μg of protein was reduced and alkylated with 100 mM triethylammonium bicarbonate (TEAB) buffer, 10 mM tris (2-carboxyethyl) phosphine, and 40 mM iodoacetamide, incubated for 40 min at room temperature in the dark. After centrifugation at 4°C for 20 min, the precipitates were dissolved in 100 μl of 100 mM TEAB buffer and digested with trypsin overnight at 37°C. The resulting peptides were desalted, vacuum-dried, and quantified. Peptide analysis was performed using an VanquishNeo system coupled with an Orbitrap Astral mass spectrometer (Thermo, USA) at Majorbio Bio-Pharm Technology Co. Ltd. (Shanghai, China). The ES906 column (150 μm × 15 cm, Thermo, USA) was used with solvent A (water with 2% ACN and 0.1% formic acid) and solvent B (water with 80% ACN and 0.1% formic acid). Protein identification was performed using Spectronaut software (Version 18) with stringent parameters [Protein false discovery rate (FDR) ≤ 0.01, Peptide FDR ≤ 0.01], considering only proteins with at least one unique peptide. Bioinformatic analysis of proteomic data was performed with the Majorbio Cloud platform. Differentially expressed proteins (DEPs) were identified using the R package “t-test” with thresholds of |log_2_ fold change| ≥ 1.0 and BH-adjusted *P* < .05. Functional annotation and enrichment analysis of identified proteins were performed using GO terms and KEGG pathways.

### Growth assays of *R. solanacearum* with *S. maltophilia* and *Bacillus* co-culture supernatants

The impact of *S. maltophilia* P373 on the antimicrobial activity of three *Bacillus* strains was evaluated through co-culture experiments (Fig. 1B, a). Varying ratios of *S. maltophilia* P373 were inoculated alongside each *Bacillus* strain to evaluate dose-dependent effects. Briefly, after overnight cultivation, the cells of each strain were harvested and resuspended in sterile water to an initial density of ~10^7^ CFU ml^−1^. Subsequently, *S. maltophilia* P373 and each *Bacillus* strain were mixed at inoculum gradients of 20, 40, 80, 160, 320, and 640 μl, with sterile water added to ensure the total volume was consistently maintained at 1280 μl. In total, 126 cell suspensions (3 *Bacillus* species × 6 inoculation volumes of *Bacillus* × 7 inoculation volumes of *S. maltophilia*) were inoculated into 20 ml BG mediums and incubated at 30°C for 30 h, after which the spent media were collected by centrifugation and 0.22 μm filtration. To test the activity of co-culture supernatants against *R. solanacearum*, 5 μl of the pathogen cell suspensions (OD_600_ = 0.2) was inoculated in 150 μl of fresh BG media supplemented with 50 μl of cell-free supernatant harvested from each of the co-culture systems. After 24 h of incubation at 30°C and 160 rpm, the pathogen growth was evaluated by measuring OD_600_ of the cultures.

### The effect of nutrients on the interactions between *S. maltophilia* and *Bacillus* spp.

To assess the influence of nutrients on the interspecies competition, *R. solanacearum*, *S. maltophilia* P373, and each *Bacillus* strain were co-inoculated into 20 ml of full strength (high nutrient) or 1/10 strength (low nutrient) BG medium at an initial density of ~10^6^ CFU ml^−1^. The co-cultures were incubated at 30°C with shaking (150 rpm) for 24 h and then diluted with fresh medium at a ratio of 1:500 for subsequent passages (Fig. 1B, b). A total of five serial passages were performed in the high and low nutrient conditions. At the end of each passage, population levels of *Bacillus* strains and P373 were determined by dilution plating on LB agar. Additionally, the samples of supernatants were collected for inhibitive effect analysis against *R. solanacearum* PA1 as described above.

We further assessed the antagonistic effect of the co-culture supernatants against the pathogen. Briefly, 160 μl of each strain (~10^7^ CFU ml^−1^) was inoculated into both BG medium and 1/10 strength BG mediums in triplicates. Following the above procedure, the cultures were incubated and diluted into fresh media every 24 h. At the end of each incubation cycle, cell-free supernatants were collected for pathogen inhibition bioassays, while the cells were harvested and stored at −80°C for subsequent DNA extraction and 16S rRNA gene amplicon sequencing.

### Soil microcosm experiments

To explore the influence of resource availability on the interplay among the antibiotic-producing *Bacillus* species (*B. amyloliquefaciens* P224, *B. subtilis* P165, and *B. velezensis* P63), the antibiotic-degrading *S. maltophilia* P373, and the pathogen *R. solanacearum* PA1 in soil microbiomes, a soil microcosm study involving the supply of carbon resources was conducted (Fig. 1B, c). First, we determined the carbon utilization profile of each strain using 50 different carbohydrates, amino acids, and organic acids present in tomato root exudates [47]. This was conducted in 96-well plates by inoculating 5 μl of cell suspension (OD_600_ = 0.2) of each strain into 150 μl of 1/5 strength BG medium supplemented with 50 μl of 10 mM solution of a carbon source. The control treatments received an equal volume of sterile water instead of the carbon source. Each treatment had four replicates. After 24 h of incubation at 30°C, the bacterial growth was determined by measuring the OD_600_, and the C source effect was evaluated by calculating ratios between the carbon source-treated and control groups. Ratio values >1.0 suggest that the C source is utilized and promotes the bacteria growth. Based on this test, six highly utilized C sources were selected for further experiments, including D-glucose, D-fructose, D-ribose, L-glutamine, L-alanine, and L-serine (Supplementary Table S2). The carbon sources were prepared as 5-, 10-, 20-, and 40-mM solutions and 100 ml of each of the solutions were thoroughly mixed into 1.0 kg of steam-sterilized soil pre-inoculated with 50 ml of bacterial suspensions (OD_600_ = 1.0) containing equal concentrations of *B. amyloliquefaciens* P224, *B. subtilis* P165, *B. velezensis* P63, *S. maltophilia* P373, and *R. solanacearum* PA1 cells. Each treatment had four replicates. The inoculated soils were transferred into 250 ml glass bottles and incubated at 30°C for 14 days to allow the community to stabilize. After the incubation, 0.5 g of soil was collected from each bottle for DNA extraction. The bacterial community composition was evaluated by 16S rRNA gene amplicon sequencing analysis, while *R. solanacearum* levels were determined by qPCR.

### Greenhouse plant assays

The impact of *S. maltophilia* P373 on the ability of *Bacillus* strains to control bacterial wilt of tomatoes caused by *R. solanacearum* was evaluated in plant experiments (Fig. 1A, b). Tomato seeds (*L. esculentum*, cultivar Hezuo 903) were surface sterilized by soaking in 5% NaClO for 5 min and then germinated in sterile Petri dishes. After 1 week, the seedlings were individually transplanted into sterile Falcon tubes containing 45 ml autoclaved vermiculite. The plants were grown in an environmental chamber under a photon flux density of 300 μmol m^−2^ s^−1^, 14 h/10 h day/night cycle, 30°C/ 26°C day/night temperature, and 70% relative humidity. The plants were watered every 3 days and irrigated with 10 ml of 50% Hoagland’s nutrient solution once a week. Fifteen days after the transplantation, some tubes were inoculated with 5 ml of bacterial suspension containing the three *Bacillus* strains (*B. amyloliquefaciens* P224, *B. subtilis* P165, and *B. velezensis* P63, respectively; OD_600_ = 1.0), 5 ml of the antibiotic degrader *S. maltophilia* P373 (OD_600_ = 0.5), and the same volume of the pathogen *R. solanacearum* PA1 (OD_600_ = 1.0) were added to the substrate. The control tubes were inoculated with the three *Bacillus* species, *R. solanacearum*, and 5 ml of sterile water instead of *S. maltophilia*. Eight biological replicates of the treatment and control were used in the experiment. The symptoms of bacterial wilt were assessed daily and recorded according to the following disease index [48]: 0, no wilted leaves; 1, partial leaves wilting; 2, all leaves are wilted. Daily disease incidence was calculated for 10 days post-inoculation using the following formula [49]: disease incidence (%) = (the number of diseased plants in each disease index × disease index) / (total number of plants × the highest disease index) × 100. The disease development was evaluated using the area under the disease progress curve (AUDPC) method [50], calculated by trapezoidal integration of temporal disease incidence data according to the following formula: AUDPC = *Σ* [0.5 × (*D_n_* + *D*_*n* − 1_)] × (*T_n_* − *T*_*n* − 1_), where *D_n_* and *D*_*n* − 1_ are disease incidence at day *T_n_* and *T*_*n* − 1_, respectively.

Further, we assessed the influence of C source availability on the severity of tomato wilt disease (Fig. 1B, d). The seedlings were prepared as described above, and 15 days after the transplantation, each pot was inoculated with 5 ml of bacterial suspension (OD_600_ = 1.0) containing equal numbers of *B. amyloliquefaciens* P224, *B. subtilis* P165, and *B. velezensis* P63 cells, along with 5 ml of *S. maltophilia* P373 suspension (OD_600_ = 1.0). Subsequently, 5 ml of a carbon source solution were added. Two concentrations, 5 and 40 mM, were used in the experiment to create the low and high nutrient conditions, while controls received 5 ml of sterile water. Each treatment contained 15 biological replicates. Three days after the treatment, the pathogen *R. solanacearum* PA1 (5 ml, OD_600_ = 1.0) was inoculated into the substrates. The disease index and incidence of wilt symptoms were recorded daily for 2 weeks following the pathogen addition.

### Quantification of *R. solanacearum* by qPCR

The pathogen abundance in soil microcosm and field experiments was quantified by qPCR with a primer set targeting the *fliC* gene of *R. solanacearum* (forward primer: 5′-GAACGCCAACGGTGCGAACT-3′; reverse primer: 5′-GGCGGCCTTCAGGGAGGTC-3′) [51]. Briefly, the amplifications were performed in 20-μl reactions containing 2 μl of primers, 7 μl of ddH_2_O, and 10 μl of SYBR Green I (Sigma, Shanghai, China). The cycling program included 95°C for 1 min; 40 cycles of 95°C for 15 s, 60°C for 15 s, and 72°C for 30 s; followed by 95°C for 5 s and 60°C for 1 min; with a final ramp to 95°C for 15 s. The SYBR fluorescence was measured at the end of each amplification cycle. Three technical replicates were included for each sample.

### 16S rRNA gene amplicon sequencing and bioinformatics analyses

Total DNA was extracted from soil samples with the Fast DNA Spin kit (MP Biomedicals, Shanghai, China) and used in amplicon sequencing targeting the V3–V4 and V1–V9 regions of 16S rRNA. The V3–V4 region of the 16S rRNA gene was amplified using primers 338F (5′-ACTCCTACGGGAGGCAGCA-3′) and 806R (5′-GGACTACHVGGGTWTCTAAT-3′) [52], while the V1–V9 region was amplified using primers 27F (5′-AGAGTTTGATCMTGGCTCAG-3′) and 1492R (5′-ACCTTGTTACGACTT-3′) [53]. The libraries were prepared and sequenced on a MiSeq System (Illumina) by Personalbio (Shanghai, China). The sequence data were analysed with QIIME2 software [54]. The DADA2 plugin was used for filtering, denoising, merging, and removing chimera from the raw data [55]. Subsequently, nonsingleton amplicon sequence variants were inferred from the remaining sequences and were classified taxonomically using the SILVA database (Version 138.1) as the reference [56].

### Statistical analysis

The data are presented as the mean ± SD. Student’s t-test was used to evaluate the differences between two sample groups, while one-way analysis of variance (ANOVA) was used to analyse the differences between multiple groups. Principal coordinates analysis and permutational multivariate ANOVA tests were conducted to examine the differences in soil community composition using the “vegan” [57] and “pairwiseAdonis” [58] package in R Version 4.0.4 (R Core Team, Vienna, Austria). Co-occurrence networks were constructed for field diseased and healthy tomato rhizosphere soils using the “PlotWeb” function of the “DescTools” [59] package in R. Pairwise associations among the top 20 dominant rhizosphere genera were calculated using Spearman correlation coefficients. The resulting *P* values were adjusted for multiple testing using the Benjamini–Hochberg FDR procedure, with only statistically significant associations (adjusted *P* values <.05) retained for network visualization and subsequent analysis. The relationships between key rhizosphere taxa and the pathogen *R. solanacearum* were assessed using both linear and log-linear regression models implemented with the “lm” function in R, with optimal model selection based on minimized Akaike Information Criterion and maximized Wherry-adjusted coefficient of determination (*R*^2^).

## Results

### Health of field tomato plants is correlated with the interactions between *Stenotrophomonas*, *Bacillus* spp., and the plant pathogen *R. solanacearum*

**Figure 2:**
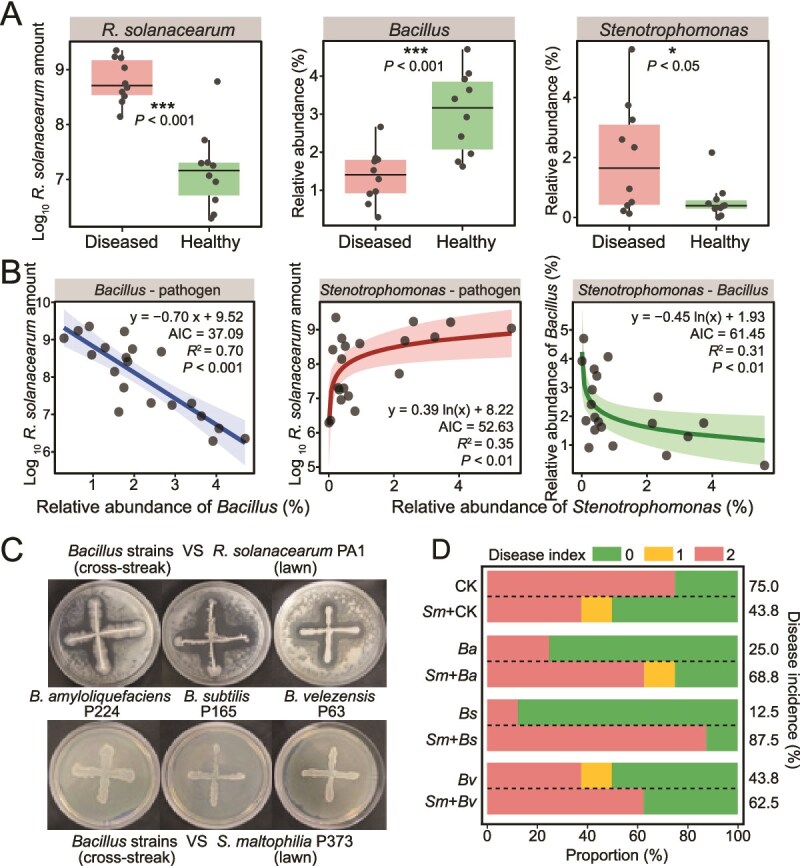
The relationships among *Stenotrophomonas*, *Bacillus*, and the invasion of plant pathogen *Ralstonia solanacearum*. (A) The abundance of *R. solanacearum*, *Bacillus*, and *Stenotrophomonas* in the rhizosphere soil of diseased and healthy plants. “^*^”, “^**^”, and “^***^” represent significant differences (Student’s t-test, *P* < .05, .01, and .001, respectively) between the diseased and healthy group. (B) the regressions of pairwise correlations between qPCR levels of *R. solanacearum* and the relative abundance of *Bacillus* and *Stenotrophomonas* in the field. (C) Plate confrontation assays to evaluate the antagonistic effect of *B. amyloliquefaciens* P224, *B. subtilis* P165, and *B. velezensis* P63 on *R. solanacearum* PA1 and *S. maltophilia* P373. (D) The disease incidence and disease index in tomato seedlings on the 10th day of the minipot experiment. “CK” represents the control group without *Bacillus* strain treatment. “*Ba,*” “*Bs,*” and “*Bv*” represent plants treated with *B. amyloliquefaciens* P224, *B. subtilis* P165, and *B. velezensis* P63, respectively. “*Sm*” represents the treatment inoculated with *S. maltophilia* P373.

In this study, we observed the occurrence of bacterial wilt disease in the tomato greenhouses (Supplementary Fig. S1A). To decipher the disease phenomenon in the field, we investigated the pathogen abundance and community structure in the diseased and healthy tomato rhizosphere. The population of causal pathogen, *R. solanacearum*, was significantly higher (*P* < .001) in the rhizosphere of diseased plants (~10^8^–10^9^ copies/g) compared with healthy plants (~10^6^–10^8^ copies/g soil; Fig. 2A). Moreover, these changes in pathogen abundance correlated with shifts in the abundance of genus *Bacillus* and *Stenotrophomonas*. In comparison to healthy plants, the rhizosphere microbiome of diseased plants had a lower abundance of *Bacillus* (*P* < .001) and a higher abundance of *Stenotrophomonas* (*P* < .05). Network analysis showed that among the top 20 dominant genera in the rhizosphere, the abundance of *Bacillus*, *Streptomyces*, *Saccharimonadales*, *Bryobacter*, *Nocardioides*, *Rhodanobacter*, and *Chujaibacter* negatively correlated with *Ralstonia*, while only *Stenotrophomonas* positively correlated with *Ralstonia* (*P* < .05) (Supplementary Fig. S1B). The regression analysis further revealed that *Bacillus* abundance was negatively correlated with *R. solanacearum* qPCR levels (*R*^2^ = 0.70, *P* < .001; Fig. 2B), whereas *Stenotrophomonas* abundance positively correlated with the pathogen (*R*^2^ = 0.35, *P* < .01). Furthermore, a significant negative relationship was observed between *Stenotrophomonas* and *Bacillus* (*R*^2^ = 0.31, *P* < .01), implying their contrasting roles in the assembly of the rhizosphere community and the development of disease. These results suggest that their interaction in the rhizosphere may determine the bacterial wilt in the tomato.

### Antibiotic-resistant *S. maltophilia* protects *R. solanacearum* from biocontrol *Bacillus* strains

To explore their interactions, we isolated *Stenotrophomonas maltophilia*, the tomato wilt pathogen *R. solanacearum*, and three strains of *Bacillus* from tomato rhizosphere of diseased tomato plants. Based on our plate assays and supernatant culture experiments showing no direct positive interaction between *S. maltophilia* and *R. solanacearum* (Supplementary Figs S2 and S3), we hypothesized that *Stenotrophomonas* somehow interferes with beneficial *Bacillus* spp. and promotes the pathogen invasion success and then induced bacterial wilt in tomatoes. We initiated this study by verifying the antagonistic activity of *B. amyloliquefaciens* P224, *B. subtilis* P165, and *B. velezensis* P63 against the tomato wilt pathogen *R. solanacearum* PA1. All three *Bacillus* strains demonstrated potent antagonism evidenced by the clearance zones around their colonies in plate confrontation assays (Fig. 2C). In contrast, no antagonistic activity was observed on plates with *S. maltophilia* P373, indicating its resistance to the selected *Bacillus* spp.

We further tested whether *S. maltophilia* P373 interferes with the ability of *B. amyloliquefaciens* P224, *B. subtilis* P165, and *B. velezensis* P63 to control bacterial wilt in tomatoes caused by *R. solanacearum*. Treating tomato seedlings with the three *Bacillus* strains significantly lowered the disease incidence to 25%, 12.5%, and 43.8% compared with 75% in the untreated control (Fig. 2D and Supplementary Fig. S4). The presence of *S. maltophilia* interfered with the biocontrol activity of the *Bacillus* strains, significantly increasing disease incidence to 68.8%, 87.5%, and 62.5%. When *S. maltophilia* was inoculated alone, the incidence of wilting decreased to 43.8% compared with the control, indicating the biocontrol effect against bacterial wilt. Collectively, these observations suggest that *S. maltophilia* promotes the disease by interfering with protective activity of the *Bacillus* strains and that this phenomenon occurs only in the presence of antagonistic bacteria.

### *S. maltophilia* inactivates lipopeptide antibiotics produced by *Bacillus* species

**Figure 3:**
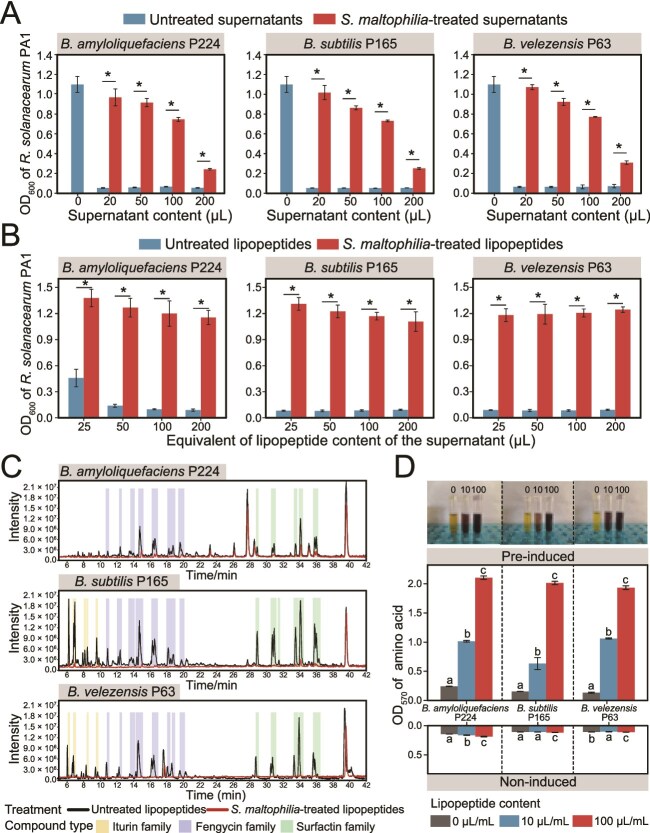
*Stenotrophomonas maltophilia* reduces the toxicity of *Bacillus* culture supernatants against *R. solanacearum*. (A) Growth of *R. solanacearum* at 24 h in the presence of increasing volumes of *Bacillus* culture supernatants untreated or treated with *S. maltophilia*. All cultures were adjusted to a final volume of 200 μl with fresh BG medium. (B) Growth of *R. solanacearum* at 24 h in BG medium supplemented with different concentrations of LP extracts from strains P224, P165, and P63. Asterisks indicate significant differences between the untreated and treated groups (Student’s t-test, *P* < .05). (C) Total ion chromatograms of LPs extracted from culture supernatants *B. amyloliquefaciens* P224, *B. subtilis* P165, and *B. velezensis* P63, before and after exposure to *S. maltophilia*, respectively. (D) The release of amino acids after treatment of *Bacillus* LPs with cell extracts from LP-induced (pre-induced) or untreated (noninduced) *S. maltophilia*. Different letters represent significant differences among treatments based on one-way ANOVA (*P* < .05).

To unravel the interaction mechanisms behind the biocontrol interference by *S. maltophilia*, we evaluated the capacity of this organism to inactivate metabolites produced by *Bacillus* strains. As expected, culture supernatants of *B. amyloliquefaciens* P224, *B. subtilis* P165, and *B. velezensis* P63 significantly inhibited the growth of *R. solanacearum* (Fig. 3A). In contrast, the same supernatants exposed to *S. maltophilia* exhibited significantly reduced pathogen inhibition.

Given that nonribosomal LPs are the primary biocidal substance responsible for the antagonistic activity of *Bacillus* species [60, 61], we extracted LPs from each *Bacillus* strain and evaluated their bioactivity against *R. solanacearum* and *S. maltophilia*. The *in vitro* assays showed that LP extraction substantially diminished the toxicity of *Bacillus* supernatants against the pathogen *R. solanacearum* (Supplementary Fig. S5). *Ralstonia solanacearum* was sensitive to LP treatments, with the minimum inhibitory concentrations of LP preparations from *B. amyloliquefaciens* P224, *B. subtilis* P165, and *B. velezensis* P63 being 1/4, 1/32, and 1/16 of their pre-extraction culture concentrations, respectively (Supplementary Fig. S6A). In contrast, *S. maltophilia* P373 was more resistant than *R. solanacearum* and was completely inhibited by 2, 1/4, and 1/4 times higher concentrations of LP extracts from *B. amyloliquefaciens* P224, *B. subtilis* P165, and *B. velezensis* P63, respectively. Treating *Bacillus* culture supernatants with *S. maltophilia* P373 significantly reduced the amount of LP precipitates (Supplementary Fig. S6B). The addition of the LP extracts of *S. maltophilia*-treated supernatants to *R. solanacearum* cultures resulted in increased pathogen biomass in comparison to untreated controls (Fig. 3B).

To investigate whether *S. maltophilia* P373 degrades LPs, the extracts of *Bacillus* culture supernatants treated by *S. maltophilia* were screened by ESI-TOF-MS for the presence of iturin, fengycin, and surfactin compounds which account for the main antibiotics of *Bacillus*. Fengycin- and surfactin-like metabolites were detected in LP extracts of all three *Bacillus* species, whereas iturin-like compounds were observed only in *B. subtilis* P165 and *B. velezensis* P63 (Fig. 3C and Supplementary Tables S3–S5). Subsequent MS/MS analysis identified four primary LP families: iturin A, fengycin A, fengycin B, and surfactin A (Supplementary Figs S7–S10). Within each family, homologs exhibited a 14-Da mass difference, reflecting fatty acid chain length variation via CH₂ unit addition or loss. Crucially, the peak areas of iturin, fengycin, and surfactin decreased significantly after treatment with *S. maltophilia*, suggesting that this organism can break down LP antibiotics.

The treatment of LP preparations with cellular extract of *S. maltophilia* P373 resulted in the release of degraded amino acid products identified by the ninhydrin reaction (Fig. 3D). The quantity of the degradation products increased proportionally to the initial LP content. Interestingly, prior exposure to *Bacillus* LPs significantly increased the capacity of *S. maltophilia* P373 cell extracts to break down iturin, fengycin, and surfactin, suggesting induction of hydrolytic enzymes in response to environmental antibiotic stimuli. Moreover, the quantitative LC–MS/MS analysis showed the treatment with *S. maltophilia* cellular extract significantly increased the levels of 11 amino acids within the cyclic peptide moiety of LPs (Supplementary Fig. S11), further demonstrating the enzymatic LP degradation by *S. maltophilia*.

### *Bacillus* lipopeptides induce differential gene and protein expression in *S. maltophilia*

**Figure 4:**
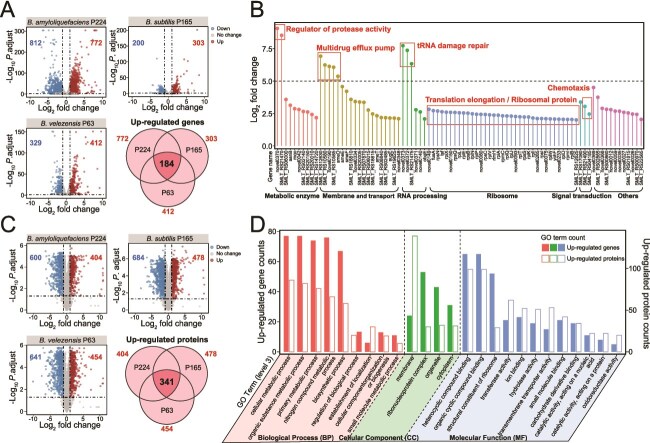
Transcriptomic and proteomic responses of *S. maltophilia* to LPs of *B. amyloliquefaciens* P224, *B. subtilis* P165, and *B. velezensis* P63. (A) Volcano plots of DEGs and Venn diagram of genes upregulated in response to LP extracts of *Bacillus* spp. (B) The COG annotation of *S. maltophilia* highly up-regulated genes (Log_2_ fold change >2.0) induced in response to LPs of all three *Bacillus* species. (C) Volcano plots of DEPs and Venn diagram of proteins upregulated in response to LP extracts of *Bacillus* spp. (D) The GO terms (level 3) of *S. maltophilia* genes and proteins induced in response to LPs of all three *Bacillus* species.

We also integrated transcriptomic and proteomic analyses to gain insight into the molecular responses of *S. maltophilia* to LPs of *Bacillus* spp. The exposure of *S. maltophilia* P373 to LP extracts of *B. amyloliquefaciens* P224, *B. subtilis* P165, and *B. velezensis* P63 resulted in significant upregulation of 772, 303, and 412 genes, respectively (|log_2_ fold change| ≥ 1.0 and *P*-adjust <.05) (Fig. 4A). Among these, 184 genes were commonly regulated under all three *Bacillus* LP treatments, with 85 genes showing an average log_2_ fold change >2.0. Based on Clusters of Orthologous Groups (COG) functional annotation, these highly upregulated genes are primarily associated with metabolic enzymes (e.g. regulator of protease activity), membrane and transport (e.g. multidrug efflux pump), RNA processing (e.g. tRNA damage repair), ribosome (e.g. translation and ribosomal proteins), and signal transduction (e.g. chemotaxis) (Fig. 4B and Supplementary Table S6). At the same time, 404, 478, and 454 proteins were significantly upregulated (|log_2_ fold change| ≥ 1.0 and *P*-adjust <.05), including 341 proteins induced by LPs of all three *Bacillus* species (Fig. 4C), among which 18 proteins were associated with peptidase and hydrolase functions (Supplementary Table S7). GO term (level 3) annotation revealed that the commonly up-regulated 184 genes and 341 proteins shared similar functional categories. These include cellular metabolic process, organic substance metabolic process, primary metabolic process, and nitrogen compound metabolic process under Biological Process (BP); membrane under Cellular Component (CC); and heterocyclic compound binding and organic cyclic compound binding under Molecular Function (MF) (Fig. 4D).

**Figure 5:**
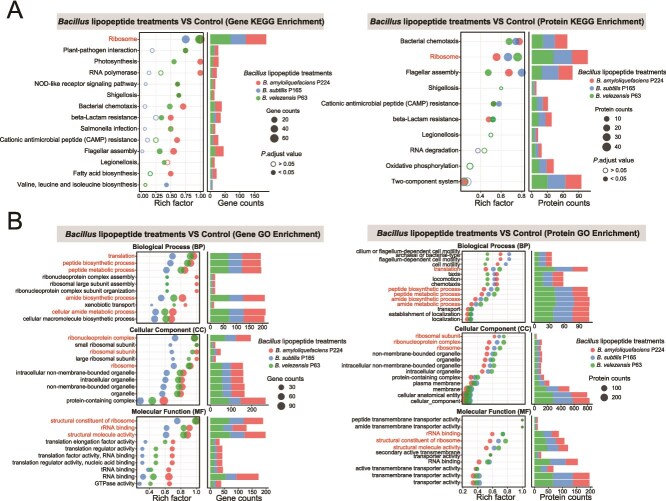
Gene and protein enrichment analysis of KEGG pathways (A) and GO terms (B) perturbed by the exposure of *S. maltophilia* to LP extracts from strains P224, P165, and P63. Bubble charts (left) of GO enrichment analysis show only the terms that were significantly enriched in *S. maltophilia* across all three *Bacillus* LP treatments, and bar charts (right) indicate the number of enriched genes or proteins. The pathways or terms highlighted indicate those that were co-enriched in both transcriptomic and proteomic profiling.

The KEGG pathway enrichment analysis of *S. maltophilia* genes differentially expressed in response to LP extracts of *B. amyloliquefaciens* P224, *B. subtilis* P165, and *B. velezensis* P63 revealed significant (*P* < .05) enrichment of ribosomes (Rich factors of 1.0, 0.75, and 0.99). Furthermore, *S. maltophilia* proteins were enriched in pathways of ribosomes (0.56, 0.67, and 0.76), bacterial chemotaxis (0.77, 0.74, and 0.68), flagellar assembly (0.68, 0.80, and 0.48), cationic antimicrobial peptide (CAMP) resistance (0.53, 0.58, and 0.53), and beta-lactam resistance (0.48, 0.52, and 0.52) (Fig. 5A). GO enrichment analysis of the three *Bacillus* LP treatments identified 65 shared terms for genes (only the top 30 enriched shown) and 39 shared terms for proteins (Fig. 5B). In the BP category, significantly enriched terms including translation, peptide biosynthetic process, peptide metabolic process, amide biosynthetic process, and amide metabolic process were identified in both transcriptomic and proteomic results, all supported by the highest counts of upregulated genes and proteins. Within the CC and MF categories, ribosomal structure and functions, such as ribonucleoprotein complex, the ribosomal subunit, ribosome, structural constituent of ribosomes, rRNA binding, and structural molecule activity, were overrepresented in both gene and protein enrichment results. Additionally, all proteins associated with the molecular functions of peptide transmembrane transporter activity and amide transmembrane transporter activity were upregulated in *S. maltophilia* under all three *Bacillus* LP treatments.

### *S. maltophilia* interferes with the ability of *Bacillus* to control *Ralstonia* wilt in a density-dependent manner and is regulated by nutrient availability

**Figure 6:**
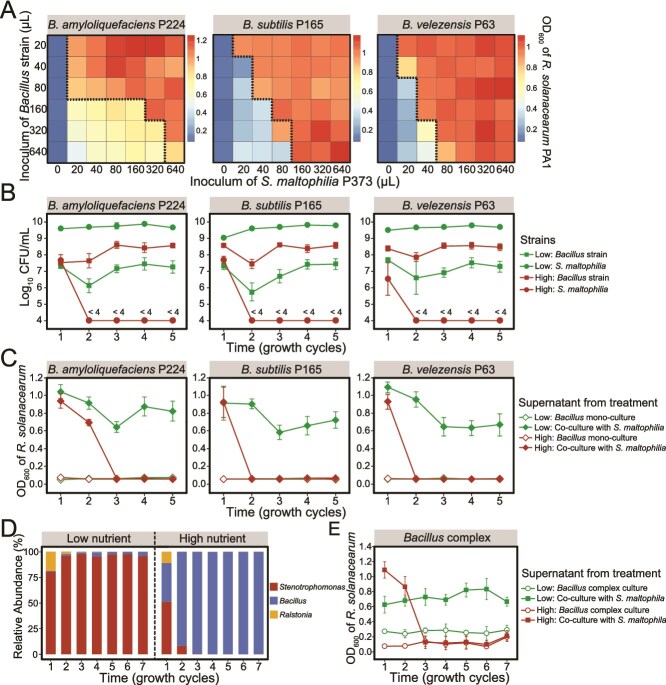
Resource availability influences the balance between the biocontrol *Bacillu*s and *S. maltophilia* and affects population levels of the *R. solanacearum* pathogen. (A) Heatmap depicting the growth of *R. solanacearum* at 24 h in the presence of varying amounts of supernatant from co-cultures seeded with different ratios of *S. maltophilia* and *Bacillus* species. Dashed lines represent the thresholds that distinguish the toxic and nontoxic conditions for *R. solanacearum*. (B) the dynamics of *S. maltophilia* and *Bacillus* spp. passaged in mixed culture under the conditions of low and high resource availability. (C) The serial passage experiment illustrating the effect of supernatants from *B. amyloliquefaciens* P224, *B. subtilis* P165, or *B. velezensis* P63 cultures or their combinations with *S. maltophilia* on the growth of *R. solanacearum* at 24 h. (D) the dynamics of *R. solanacearum*, *S. maltophilia*, and *Bacillus strains* in mixed cultures under conditions of low and high resource availability. (E) the effect of supernatants from *Bacillus* complex cultures or their combinations with *S. maltophilia* on the growth of *R. solanacearum* at 24 h. the supernatants were generated under conditions of low and high resource availability.

We further assessed the impact of bacterial interactions on *R. solanacearum* PA1 by evaluating its growth in supernatants of co-cultures of *S. maltophilia* P373 and *Bacillus* strains. The heatmap results indicated that the toxicity of co-culture supernatants to *R. solanacearum* depended on the initial inoculum density of *S. maltophilia* P373 (Fig. 6A). Treatment of the co-culture supernatants with higher populations of *S. maltophilia* led to lower inhibition and higher biomass of *R. solanacearum*. However, the effect was not linear, upon reaching a certain threshold, a further increase in the inoculum of *S. maltophilia* P373 did not result in the increased growth of *R. solanacearum* (Supplementary Fig. S12). This finding aligns with the relationships between *S. maltophilia* and *R. solanacearum* observed in field experiments, emphasizing a threshold effect on their interactions.

Recognizing that the interactions between biocontrol *Bacillus* species and *S. maltophilia* P373 influence the environmental toxicity against *R. solanacearum*, we proposed a resource regulation strategy, highlighting resource availability as a key factor in shaping bacterial interactions [40]. We explored its influence on the interaction between the *Bacillus* species and *S. maltophilia* P373. The co-culture assays revealed that low nutrient conditions favored the growth of *S. maltophilia*, while *Bacillus* spp. thrived under high nutrient availability (Fig. 6B). Furthermore, the supernatant testing demonstrated that higher nutrient availability diminishes the capacity of *S. maltophilia* to interfere with the ability of *Bacillus* cultures to control the growth of *R. solanacearum* as assessed by qPCR (Fig. 6C). These findings agreed with the results of 16S-based amplicon profiling, showing that under high nutrient conditions, *Bacillus* dominated mixed cultures, while the levels of *S. maltophilia* decreased to <1% after the second passage (Fig. 6D). In contrast, *S. maltophilia* dramatically outcompeted *Bacillus* under low nutrient conditions. Finally, *R. solanacearum* exhibited a higher relative abundance in lower nutrient conditions and exhibited lower growth in the supernatant of high nutrient co-cultures (Fig. 6D, E), suggesting a negative impact of high resources on the protection provided by *S. maltophilia*.

**Figure 7:**
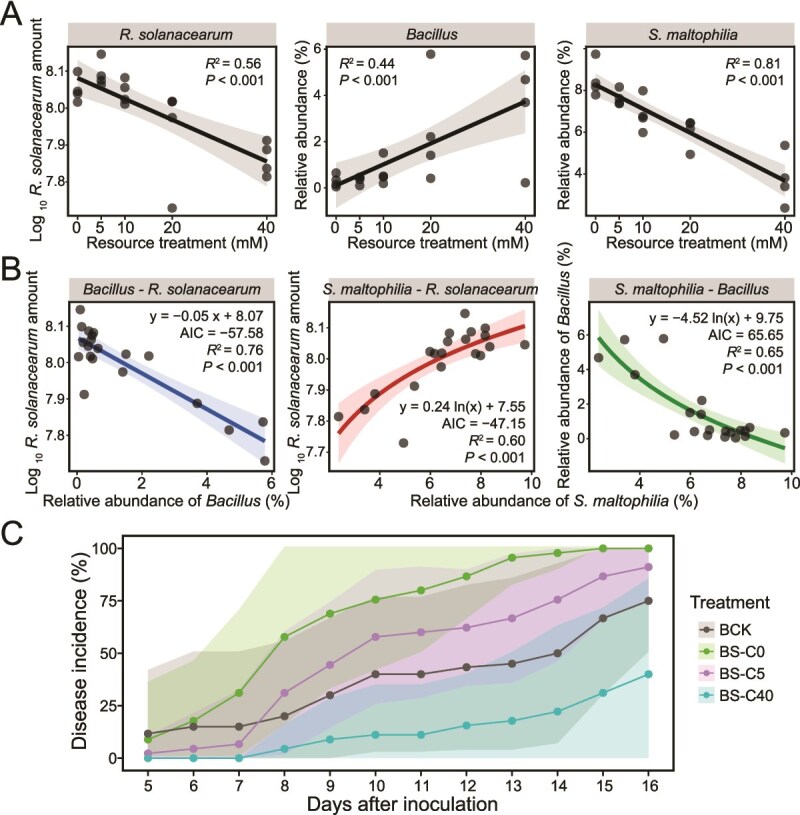
Carbon source amendments improve the ability of *Bacillus* spp. to compete with *S. maltophilia* in soil microcosms and control the bacterial wilt of tomatoes caused by *R. solanacearum*. (A) The response of *R. solanacearum*, *Bacillus* spp., and *S. maltophilia* to soil inoculation with different amounts of carbon sources. (B) Regression plots showing the pairwise correlations between the population levels of *R. solanacearum* and the relative abundance of *S. maltophilia* or *Bacillus* spp. in the soil microcosm experiment. (C) The incidence of bacterial wilt of tomato under different carbon resource availability. “BS-C0,” “BS-C5,” and “BS-C40” represent treatments inoculated with *Bacillus* spp., *S. maltophilia*, and *R. solanacearum* and amended with 0 mM, 5 mM, or 40 mM of carbon source, respectively. “BCK” represents the control inoculated with *Bacillus* spp. and *R. solanacearum* without carbon source input. The shaded area represents the standard deviation of the disease incidence rate.

Similar results were observed in soil microcosms, where the availability of carbon sources significantly influenced the structure and composition of rhizosphere communities (Supplementary Fig. S13 and Table S8). The addition of C source increased the relative abundance of *Bacillus* (*R*^2^ = 0.44, *P* < .001) and had a significant negative effect on both *R. solanacearum* (*R*^2^ = 0.56, *P* < .001) and *S. maltophilia* (*R*^2^ = 0.81, *P* < .001) (Fig. 7A). The fitting results further confirmed the negative relationship between the relative abundance of *Bacillus* and *S. maltophilia* under varying resource availability treatments (*R*^2^ = 0.65, *P* < .001) (Fig. 7B). Taken collectively, these results identify nutrient availability as a key factor influencing the ability of beneficial *Bacillus* species to compete with LP-degrading strains of *S. maltophilia*.

The regulation of nutrient availability on the interaction among three species and such induced bacterial wilt was investigated with tomato seedling assays. It demonstrated that an increase in carbon source input results in a substantial reduction in the incidence of bacterial wilt disease (Fig. 7C). On the 14th day after pathogen inoculation, the disease incidence in the treatment inoculated with *Bacillus* strains and *S. maltophilia* P373 (BS-C0) reached 97.8%, compared to 50% observed in the control group inoculated only with *Bacillus* spp. (BCK). However, the disease incidence dropped to 75.6% in the low-level C source treatment (BS-C5) and further decreased to only 22.2% under high-level C source input (BS-C40). These findings collectively highlight the importance of nutrient availability in modulating rhizosphere microbial interactions and significantly enhancing the effectiveness of *Bacillus* biocontrol agents.

## Discussion

In this work, we characterized a tripartite bacterial model system consisting of an antibiotic producer (*B. amyloliquefaciens* P224, *B. subtilis* P165, and *B. velezensis* P63), an antibiotic degrader (*S. maltophilia*), and a plant pathogen (*R. solanacearum*). We found that although *S. maltophilia* does not directly promote the pathogen *R. solanacearum* (Supplementary Fig. S3), it acts as a pathogen helper by attenuating the ability of *Bacillus* spp. to control the bacterial wilt of tomatoes caused by *R. solanacearum* (Fig. 2D). *Stenotrophomonas maltophilia* is found worldwide in plant-associated microbial communities [35], and this is the first study documenting the adverse impact of this common organism on the biocontrol of soilborne diseases. Specifically, we illustrated the ability of *S. maltophilia* to decreased biocontrol effectiveness by breaking down lipopeptide (LP) antibiotics produced by the beneficial strains *B. amyloliquefaciens* P224, *B. subtilis* P165, and *B. velezensis* P63 (Fig. 3). These *Bacillus* strains secrete surfactin-, iturin-, and fengycin-like LPs known for their biocontrol activity [32, 62]. These exometabolites are produced by nonribosomal peptide synthetases and share structures consisting of a cyclic peptide headgroup linked to a fatty acid chain, with structural variations arising from fatty acid chain length or specific amino acid substitutions [60]. We show here that *S. maltophilia* exhibits a previously unreported capacity to specifically inactivate three classes of *Bacillus*-derived antimicrobial LPs—surfactin, iturin, and fengycin. This novel phenotype trait resembles two recently characterized interspecies interactions: the *S. venezuelae*-mediated degradation of the *B. velezensis* lipopeptidome [29], and the cooperative inactivation and degradation of *Pseudomonas*-produced CLPs by *Rhodococcus globerulus* and *S. indicatrix* [30].

The resistance mechanism of *S. maltophilia* to *Bacillus* LP antibiotics involves efflux pumps, ribosomal adaptations, and enzymatic hydrolysis. The *S. maltophilia* transcriptomic and proteomic responses to LP extracts involved the up-regulation of transmembrane transporter activity, characterized by increased expression of the *smeD*, *smeE*, and *smeF* genes and proteins associated with an RND-type multidrug efflux pump (Fig. 4B and Supplementary Table S6). This efflux pump contributes to the intrinsic and acquired antibiotic resistance by actively expelling antimicrobials from the cellular environment [63, 64]. Another group of differentially expressed *S. maltophilia* genes and proteins encoded components of ribosomes and translational machinery (Fig. 5). Ribosomes play a vital biological role in maintaining cellular stability and establishing antibiotic resistance to adaptation to antibiotic stress conditions [65]. The over-expression of ribosomal proteins and translation elongation factors may counteract antibiotic interference by promoting ribosomal overproduction, mutations, or modifications, thereby preserving cellular function [66–68]. Furthermore, the RNA-splicing ligase gene *rtcB* was upregulated in *S. maltophilia* treated with *Bacillus* LPs. The RNA-splicing ligase functions to repair damaged tRNAs and accelerate recovery in response to antibiotic stress [69]. These mechanisms may also contribute to evolutionary enhancements in LP resistance (Fig. 3D). For the inactivation of cell-penetrating LP antibiotics, *S. maltophilia* showed up-regulation of genes and proteins associated with metabolic processes of organic substance, nitrogen compound, peptides, and amides, as well as heterocyclic compound binding and organic cyclic compound binding functions (Fig. 5B). Nonribosomal LPs often have cyclic structures and contain D-amino acids that render them resistant to many peptidases [70, 71], but the analysis of breakdown products suggests that *S. maltophilia* detoxifies LP antibiotics into free amino acids using inducible hydrolytic enzymes (Fig. 3D). Notably, genes and proteins linked to proteases and peptidases showed significant fold-changes in expression, indicating their potential role in cleaving CLPs (Fig. 4B and Supplementary Tables S6 and S7). Recent studies described a novel resistance strategy against nonribosomal LP antibiotics involving hydrolytic cleavage by D-stereospecific peptidases [72, 73]. In the future, it will be interesting to investigate if similar biochemical processes operate in *S. maltophilia.* Notably, given the structural divergence among the nonribosomal LP families, distinct enzymes, or transporters are likely involved, thus the underlying resistance mechanisms warrant systematic elucidation through gene knockout or isotopic labeling approaches.

The ability to detoxify antibiotics depends on the antibiotic concentration and cell density of *S. maltophilia* [28]. Similarly, in our study, higher concentrations of *S. maltophilia* cells resulted in an increased rate of *Bacillus* LP inactivation, providing in mixed cultures a greater level of protection for *R. solanacearum* (Fig. 6A). The detoxification effect was constrained by the density of *Bacillus* spp., which determined the rate of LP production. Consistent with *in vitro* experiments, our results revealed that the competitive interactions among *Bacillus* spp., *S. maltophilia*, and *R. solanacearum* also take place in the field, where the pathogen levels negatively and positively correlated with the abundance of *Bacillus* and *Stenotrophomonas*, respectively (Fig. 2B). Furthermore, although *Stenotrophomonas* exhibited no direct antagonistic activity against the LP-producing *Bacillus*, a significant negative correlation was observed between their populations. *Stenotrophomonas maltophilia* may mediated this competitive interaction through detoxification of *Bacillus*-derived LP antibiotics—compounds critical for rhizosphere competitiveness and colonization [60]—thereby indirectly limiting *Bacillus* spp. population. A recent 13-season tomato monoculture study also demonstrated that tomato bacterial wilt outbreaks were associated with elevated levels of antibiotic-resistant bacteria and resistance genes in soil [74]. These results raise concerns that the prevalence of antibiotic-resistant microbes and the horizontal transfer of resistance genes in the soil environment [75] may compromise both biological control efficacy and plant health. Notably, the interactions between biocontrol agents and antibiotic-resistant microbial populations exhibit nonlinear effects on pathogen invasion dynamics (Supplementary Fig. S12). This is likely governed by kinetic mechanisms involving cell density-dependent antibiotic production thresholds [76], dose-dependent inhibition effects [77], and concentration-gradient mediated resistance evolution [78]. Such exploration can provide deeper insights into the detailed mechanisms and behavioral outcomes of this tripartite interaction patterns. Moreover, our results showed that *R. solanacearum* benefits from *S. maltophilia* only in the presence of *Bacillus* spp. (Fig. 2D), collectively highlighting the highly complex and dynamic nature of interactions among antibiotic producers, antibiotic degraders, and plant pathogens. These complex interactions warrant further *in situ* investigation under field conditions, given their dependence on interdependent biological variables and environmental factors.

High resource availability can enhance bacterial metabolic capabilities and strengthen negative interspecies interactions [39, 40]. We hypothesize that under increased nutrient availability, *Bacillus* spp. gradually outcompetes *S. maltophilia* and enhance their biocontrol efficacy. The availability of carbon sources promotes LP production by *Bacillus* [32], increasing environmental antibiotic concentrations and toxicity against *R. solanacearum*. This also may slow the rate of resistance evolution and proliferation of detoxifier [79], ultimately leading to the gradual displacement of *S. maltophilia* by *Bacillus* species through intense interference competition (Fig. 6B, D). In comparison, *Stenotrophomonas maltophilia* is an *r*-strategist that actively exploits resources and niches by generating a substantial bacterial population in a short period [80]. This exploitative capability likely enables *S. maltophilia* to displace *Bacillus* spp. in co-cultures under low-nutrient conditions. The attenuation of the *S. maltophilia*-mediated pathogen protection under high-resource conditions was further validated in soil microcosms and plant assays supplemented with C sources present in root exudates (Fig. 7). In natural agricultural soil systems, a similar effect can be achieved using organic fertilizers that contain various carbon sources and microbial activity and interactions [81, 82]. Numerous studies have consistently shown that incorporating organic amendments can augment populations of biocontrol bacteria, such as *Bacillus* and *Pseudomonas*, thereby promoting disease suppression [83–85]. However, because soil antibiotic producers and degraders are highly diverse, their metabolic responses to different resource types, availability, and even supplementation modalities often lead to divergence in resource regulation efficiency and ecological interaction outcomes [41, 86, 87]. Therefore, the ongoing optimization of resource application based on strain-specific characteristics is crucial for effective biocontrol management, given its integral role in regulating the interplay between antibiotic-producing biocontrol microbes and organisms that can degrade these antibiotics.

**Figure 8:**
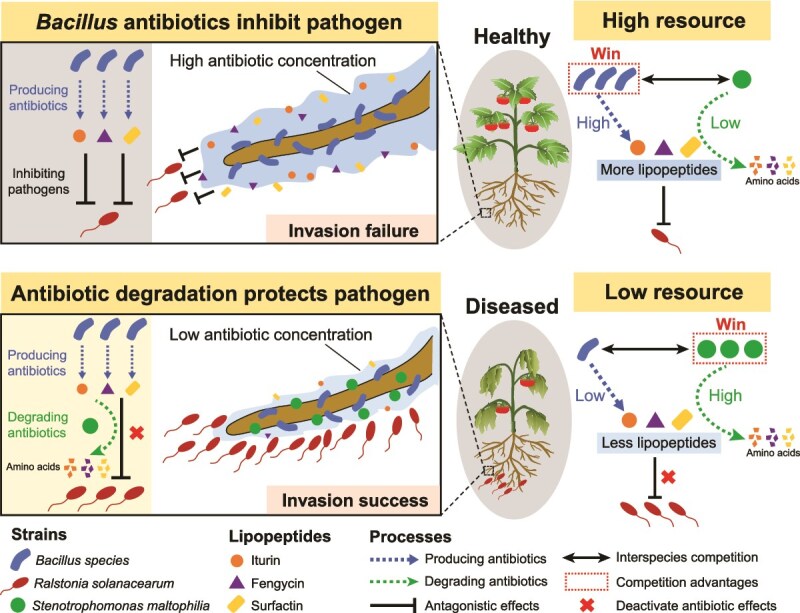
Conceptual model of the *Ralstonia* wilt promotion by the LP-degrading *S. maltophilia.* The biocontrol *Bacillus* spp. are recruited in the rhizosphere, where they promote plant health by secreting LPs that inhibit the colonization of plant roots by *R. solanacearum*. The buildup of *S. maltophilia* results in LP inactivation, thereby indirectly promoting the pathogen invasion and disease. The availability of resources influences the balance between *S. maltophilia* and *Bacillus*, ultimately affecting the disease severity. Under low resource availability, *S. maltophilia* outcompetes *Bacillus* spp. and reduces LP levels in the rhizosphere, thereby facilitating pathogen invasion. In contrast, high resource availability promotes the proliferation of *Bacillus*, which raises LP levels, leading to increased biocontrol efficacy.

In summary, this study identifies *S. maltophilia* as an organism capable of interfering with three *Bacillu*s strains by inactivating their LPs (Fig. 8). Future investigations incorporating more phylogenetically diverse *Bacillus* strains will help elucidate the broader ecological impact of this biocontrol interference. Such interspecies interactions pose a potential implementation barrier for biocontrol agents, while simultaneously offering new research avenues for plant disease management. We propose that such interactions can be manipulated by altering the amount of carbon source around plant roots and suggest that these findings warrant further investigation of this interesting and practically important biological phenomenon.

## Supplementary Material

Revised_Supplementary_Materials_(Clean)_wraf210

Supplementary_Table_wraf210

## Data Availability

All 16S rRNA gene amplicon and transcriptome sequence data are available in the National Center for Biotechnology Information (NCBI) Sequence Read Archive under BioProject accession numbers PRJNA870165, PRJNA1116630, PRJNA1116702, and PRJNA1116711. The mass spectrometry proteomics data have been deposited to the ProteomeXchange Consortium through the iProX repository under accession number PXD066400.
