# Supplementary material for: Stenotrophomonas maltophilia impedes Bacillus biocontrol of tomato wilt disease by degrading its lipopeptide antibiotics
Source: ISME J. 2025 Sep 23;19(1):wraf210. doi: 10.1093/ismejo/wraf210 (PMC12516957; doi:10.1093/ismejo/wraf210)
Supplement: Revised_Supplementary_Materials_(Clean)_wraf210 [file revised_supplementary_materials_(clean)_wraf210.docx]

**Supporting Information for**

***Stenotrophomonas maltophilia* impedes *Bacillus* biocontrol against tomato wilt disease by degrading lipopeptide antibiotics**

Junwei Peng^1,6,7^, Dmitri V. Mavrodi^4^, Jiasui Li^5^, Suhelen Egan^5^, Huanhuan Zhang^1,6,7^, Xiuli Fan^1,6,7^, Yang Liu^1,6,7^, Keke Dang^1^, Olga V. Mavrodi^4^, Qin Liu^1,7^, Yuanhua Dong^1,7^, Jiangang Li^1,2,3,6,7#^

^1^ State Key Laboratory of Soil and Sustainable Agriculture, Institute of Soil Science, Chinese Academy of Sciences, Nanjing 211135, China

^2^ College of Agricultural Science and Engineering, Hohai University, Nanjing 211100, China

^3^ College of Soil and Water Conservation, Hohai University, Nanjing 211100, China

^4^ School of Biological, Environmental, and Earth Sciences, The University of Southern Mississippi, Hattiesburg, MS, United States

^5^ Centre for Marine Science and Innovation & School of Biological, Earth and Environmental Sciences, The University of New South Wales, Sydney, NSW 2052, Australia

^6^ University of Chinese Academy of Sciences, Beijing 100049, China

^7^ Nanjing Branch of Chinese Academy of Sciences, Nanjing 211135, China

**Number of Figures in Supporting Information: 13**

**Number of Tables in Supporting Information: 8**

**Number of References in Supplementary Tables: 8**

**Figures**

**Figure S1.** Field experiments.

**Figure S2.** Plate confrontation assays to evaluate the antagonistic effect of *S. maltophilia* P373 against *R. solanacearum* PA1 (A) and *Bacillus* strains (B).

**Figure S3.** A) Growth of *R. solanacearum* at 24 h in the presence of increasing volumes (0-200 μL) of *S. maltophilia* cell-free culture supernatants. B) Growth of *S. maltophilia* at 24 h in the presence of increasing volumes (0-200 μL) of *R. solanacearum* cell-free culture supernatants.

**Figure S4.** A) Growth of tomato seedlings on the 8^th^ day of the mini-pot experiment. B) Ten-day disease progression monitoring in tomato seedlings following pathogen inoculation.

**Figure S5.** The growth of *R. solanacearum* at 24 h in the presence of increasing amounts of control *Bacillus* supernatants and lipopeptide-extracted *Bacillus* supernatants.

**Figure S6.** A) The growth of *S. maltophilia* and *R. solanacearum* for 24 h in the presence of different concentrations of lipopeptide extracts from *B. amyloliquefaciens* P224, *B. subtilis* P165, and *B. velezensis* P63. B) Isolation of lipopeptides from culture supernatants of *B. amyloliquefaciens* P224, *B. subtilis* P165*, and B. velezensis* P63.

**Figure S7.** LC-ESI-TOF-MS/MS spectra of [M+H]^+^ ions of iturin precursors.

**Figure S8.** LC-ESI-TOF-MS/MS spectra of [M+2H]^2+^ ions of fengycin precursors.

**Figure S9.** LC-ESI-TOF-MS/MS spectra of [M+2H]^2+^ ions of fengycin precursors.

**Figure S10.** LC-ESI-TOF-MS/MS spectra of [M+H]^+^ ions of surfactin precursors.

**Figure S11.** LC-MS/MS quantification of the eleven amino acids released from *Bacillus* lipopeptides following enzymatic degradation by *S. maltophilia*.

**Figure S12.** The growth of *R. solanacearum* at 24 h in the presence of varying amounts of *S. maltophilia* and *Bacillus* co-culture supernatants.

**Figure S13.** Changes in bacterial community composition in response to carbon resource availability in the soil microcosm experiment.

**Tables**

**Table S1** Summary of transcriptome sequencing data and matching rate.

**Table S2** Summary of the 50 carbon resources utilization by the strains in the soil microcosm and tomato seedling assays.

**Table S3** Assignment of lipopeptides isolated from the culture supernatant of *B. amyloliquefaciens* P224.

**Table S4** Assignment of lipopeptides isolated from the culture supernatant of *B. subtilis* P165.

**Table S5** Assignment of lipopeptides isolated from the culture supernatant of *B. velezensis* P63.

**Table S6** The categories of the 85 commonly highly up-regulated genes (average log_2_ fold change > 2.0, *P* < 0.05) in *S. maltophilia* treated with three *Bacillus* lipopeptide extracts.

**Table S7** The categories of the 341 commonly up-regulated proteins (log_2_ fold change > 1.0, *P* < 0.05) in *S. maltophilia* treated with three *Bacillus* lipopeptide extracts.

**Table S8** Permutational multivariate ANOVA (PERMANOVA) analysis of differences in bacterial community structure based on Bray-Curtis distance measures (9999 permutations).


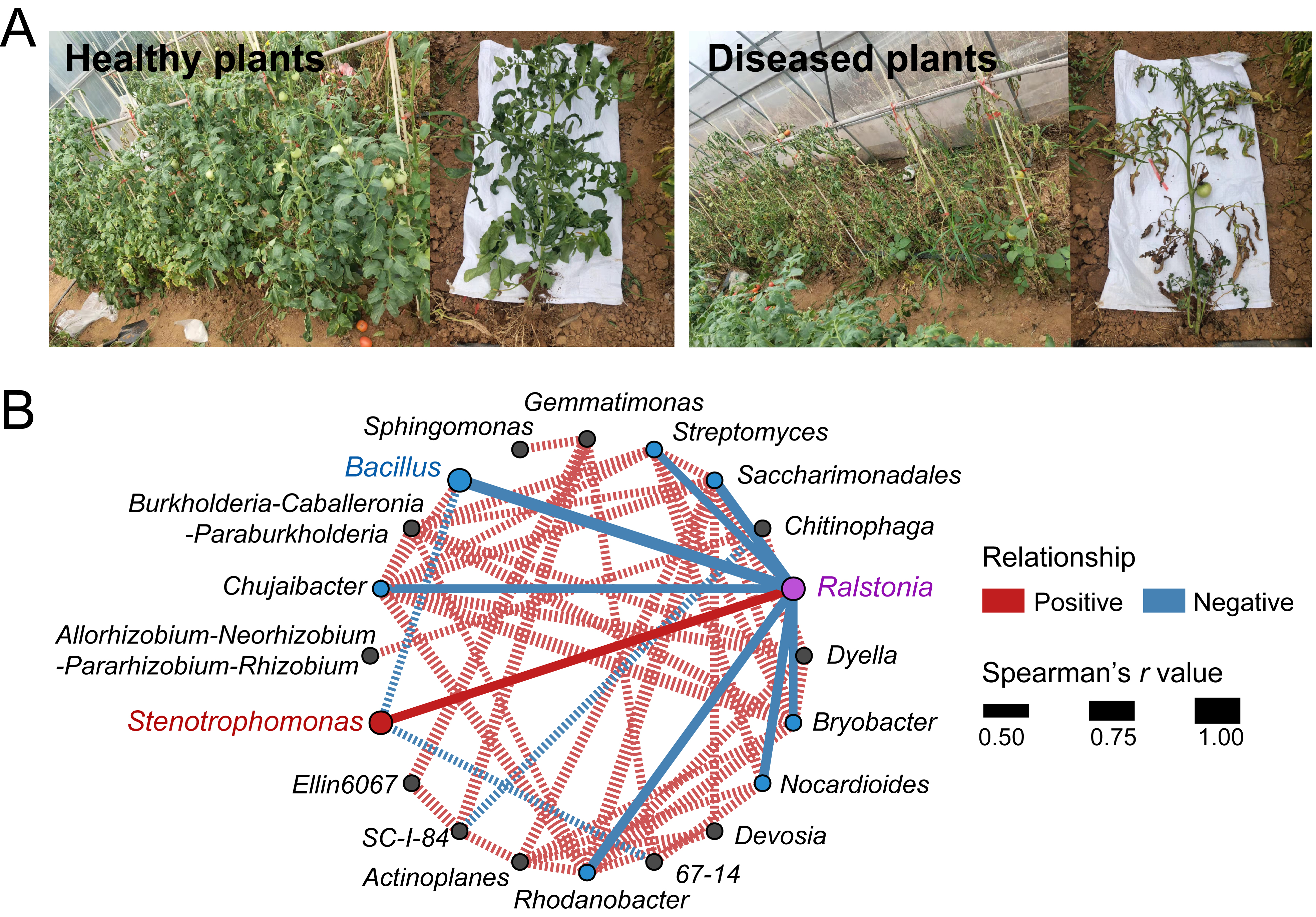


**Figure S1. Field experiments.** **A)** The appearance of healthy and diseased tomato plants. **B)** The network analysis showing pairwise Spearman’s correlations among the top dominant 20 rhizosphere genera observed in the field experiment. Correlations with values greater than 0.50 (*P* < 0.05) are shown, with solid lines indicating correlations between the *Ralstonia* pathogen and dominant taxa of rhizobacteria.


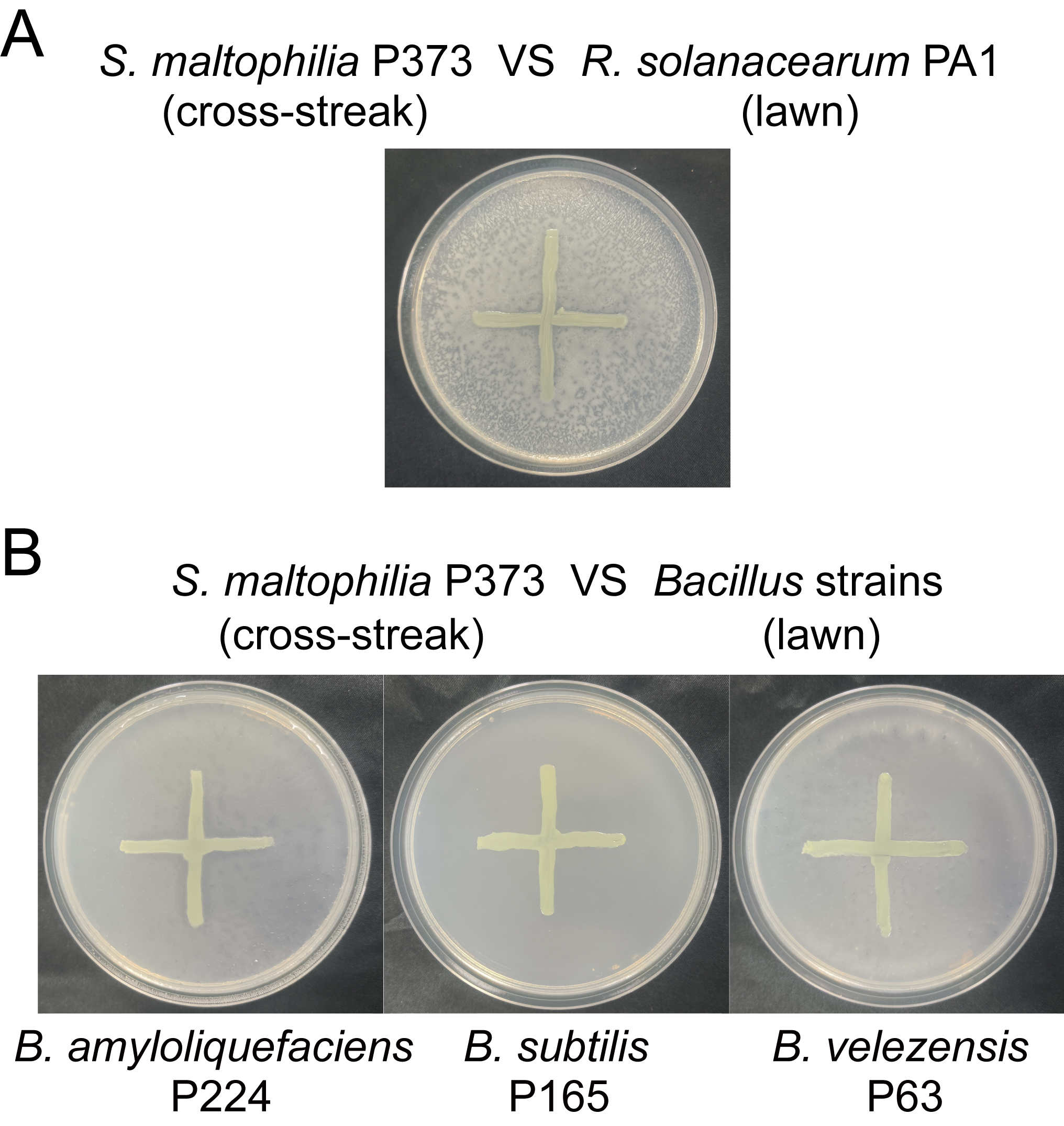


**Figure S2. Plate confrontation assays to evaluate the antagonistic effect of *S. maltophilia* P373 against *R. solanacearum* PA1 (A) and *Bacillus* strains (B).**


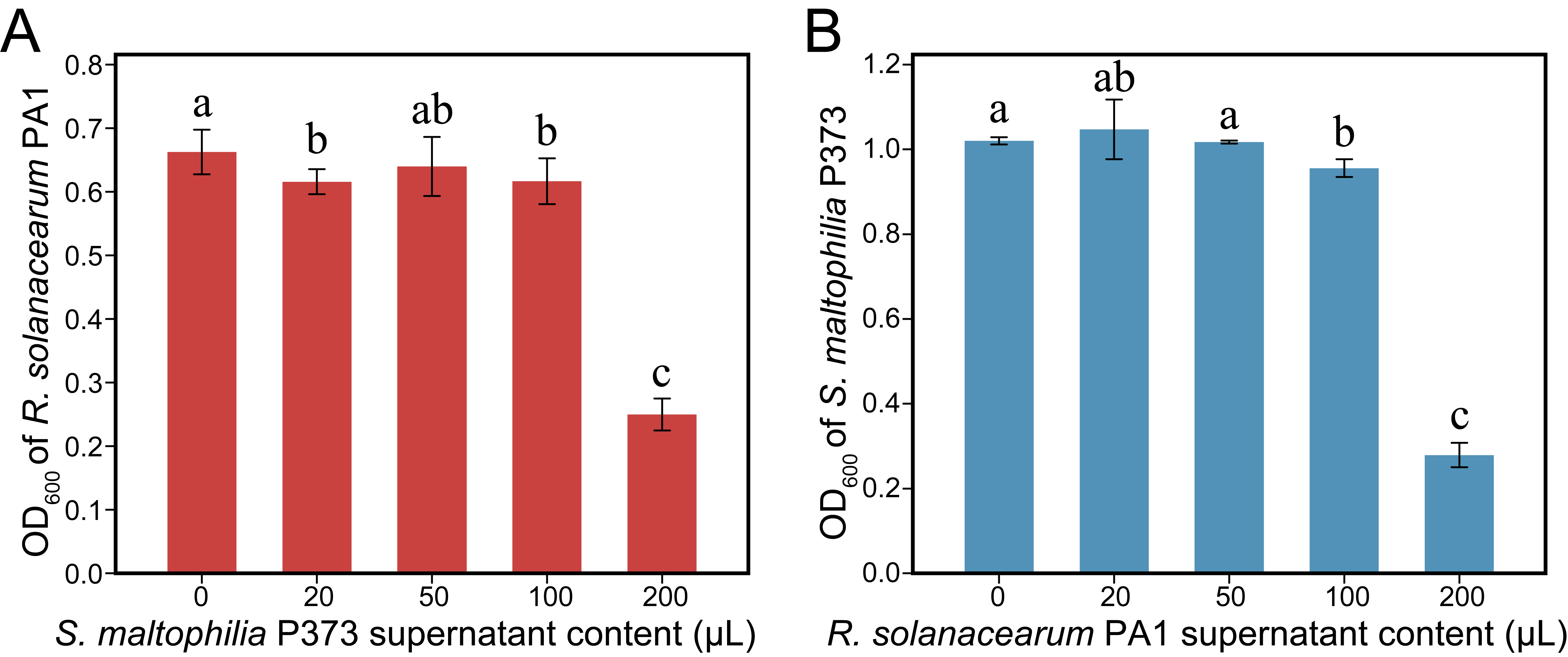


**Figure S3. A) Growth of *R. solanacearum* at 24 h in the presence of increasing volumes (0-200 μL) of *S. maltophilia* cell-free culture supernatants. B) Growth of *S. maltophilia* at 24 h in the presence of increasing volumes (0-200 μL) of *R. solanacearum* cell-free culture supernatants.** All cultures were adjusted to a final volume of 200 μL with fresh BG medium. Different letters represent significant differences among treatments based on one-way ANOVA (*P* < 0.05).

**
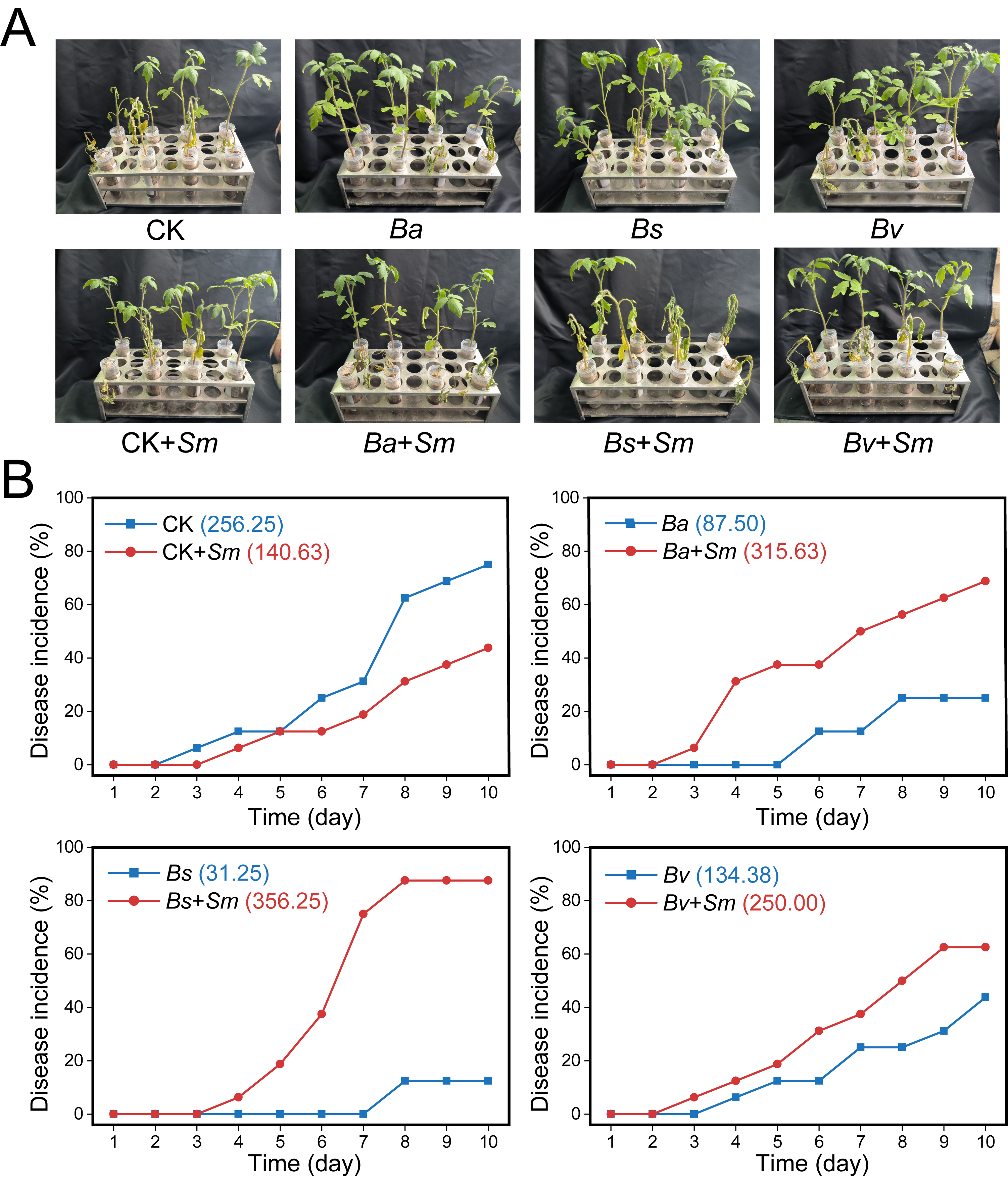
**

**Figure S4.** **A) Growth of tomato seedlings on the 8^th^ day of the mini-pot experiment. B) Ten-day disease progression monitoring in tomato seedlings following pathogen inoculation.** “CK” represents the control group without *Bacillus* strain treatment. “*Ba,*” “*Bs,*” and “*Bv*” represent plants treated with *B. amyloliquefaciens* P224, *B. subtilis* P165, and *B. velezensis* P63, respectively. “*Sm*” represents the treatment inoculated with *S. maltophilia* P373. Values in parentheses represent the area under the disease progress curve (AUDPC) calculated based on disease incidence.


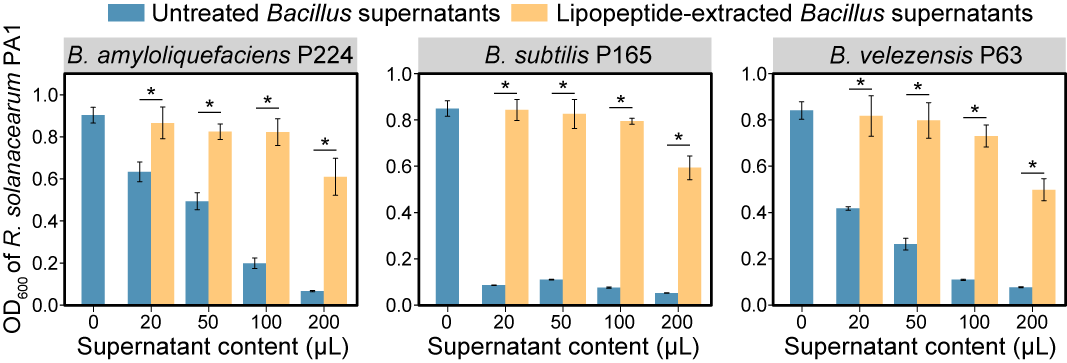


**Figure S5. The growth of *R. solanacearum* at 24 h in the presence of increasing amounts of control *Bacillus* supernatants and lipopeptide-extracted *Bacillus* supernatants.** Asterisks denote significant differences between the control and treated supernatants (Student’s t-test, *P* < 0.05).

**
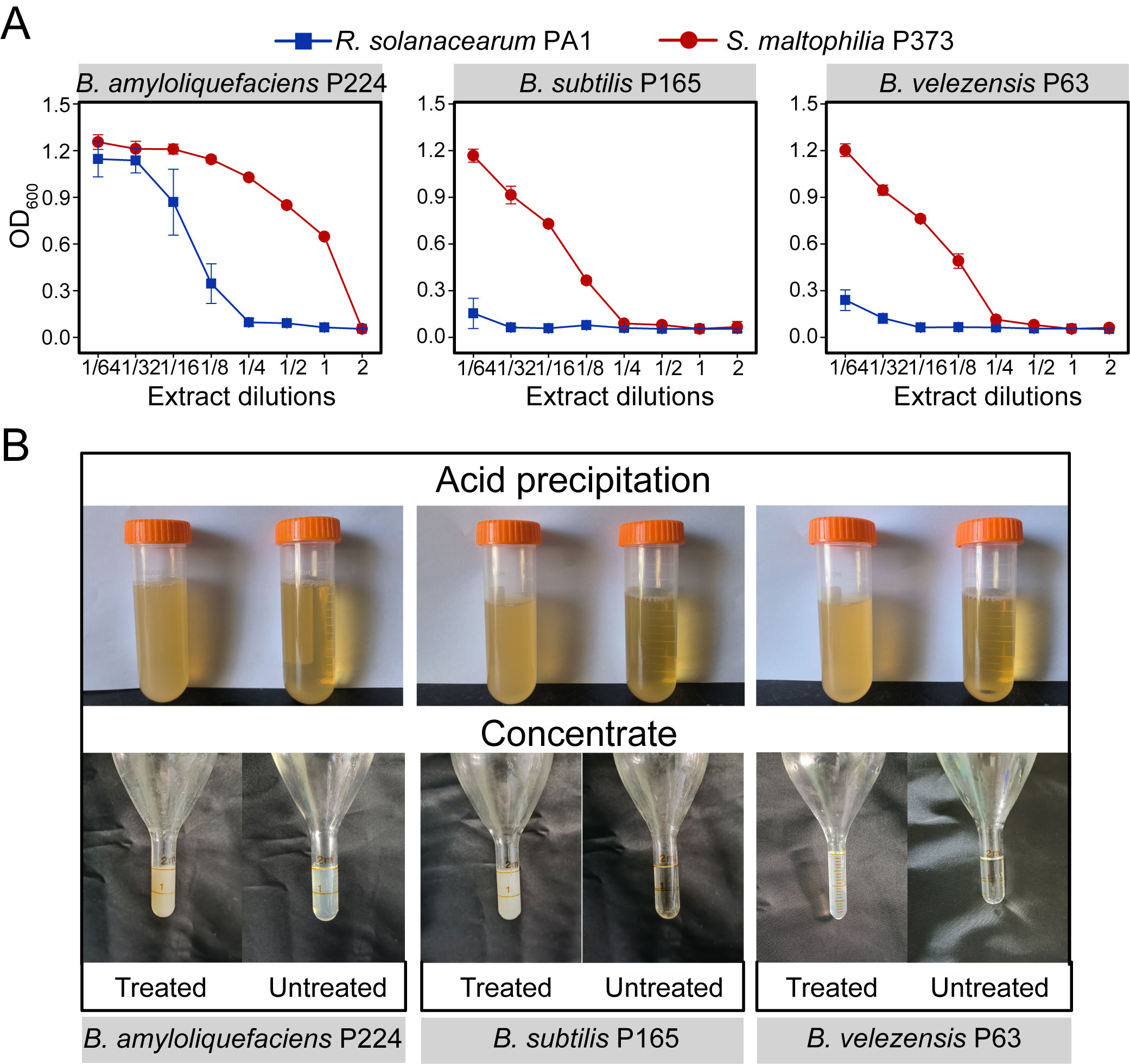
**

**Figure S6. A)** **The growth of *S. maltophilia* and *R. solanacearum* for 24 h in the presence of different concentrations of lipopeptide extracts from *B. amyloliquefaciens* P224, *B. subtilis* P165, and *B. velezensis* P63.** **B) Isolation of lipopeptides from culture supernatants of *B. amyloliquefaciens* P224, *B. subtilis* P165*, and B. velezensis* P63.** Lipopeptides were extracted by acid precipitation, dissolution in methanol, and concentrated in a rotary evaporator. The “treated” and “untreated” samples were isolated from the *Bacillus* culture supernatants treated or untreated by *S. maltophilia*.


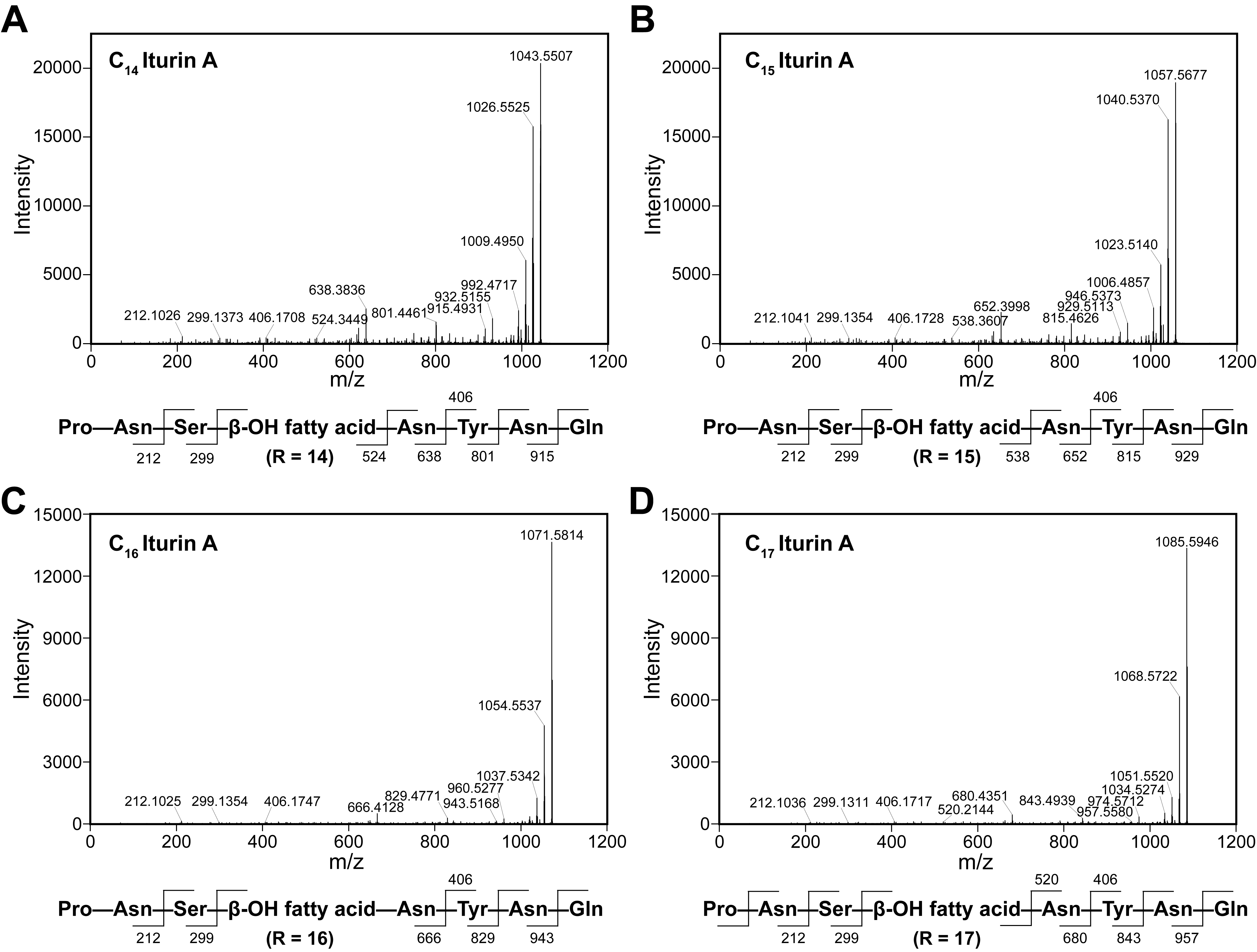


**Figure S7. LC-ESI-TOF-MS/MS spectra of [M+H]^+^ ions of iturin precursors. A)** Iturin precursor ion at *m/z* 1043, containing a C_14_ β-hydroxy fatty acid chain. **B)** Iturin precursor ion at *m/z* 1057, containing a C_15_ β-hydroxy fatty acid chain. **C)** Iturin precursor ion at *m/z* 1071, containing a C_16_ β-hydroxy fatty acid chain. **D)** Iturin precursor ion at *m/z* 1085, containing a C_17_ β-hydroxy fatty acid chain.

**
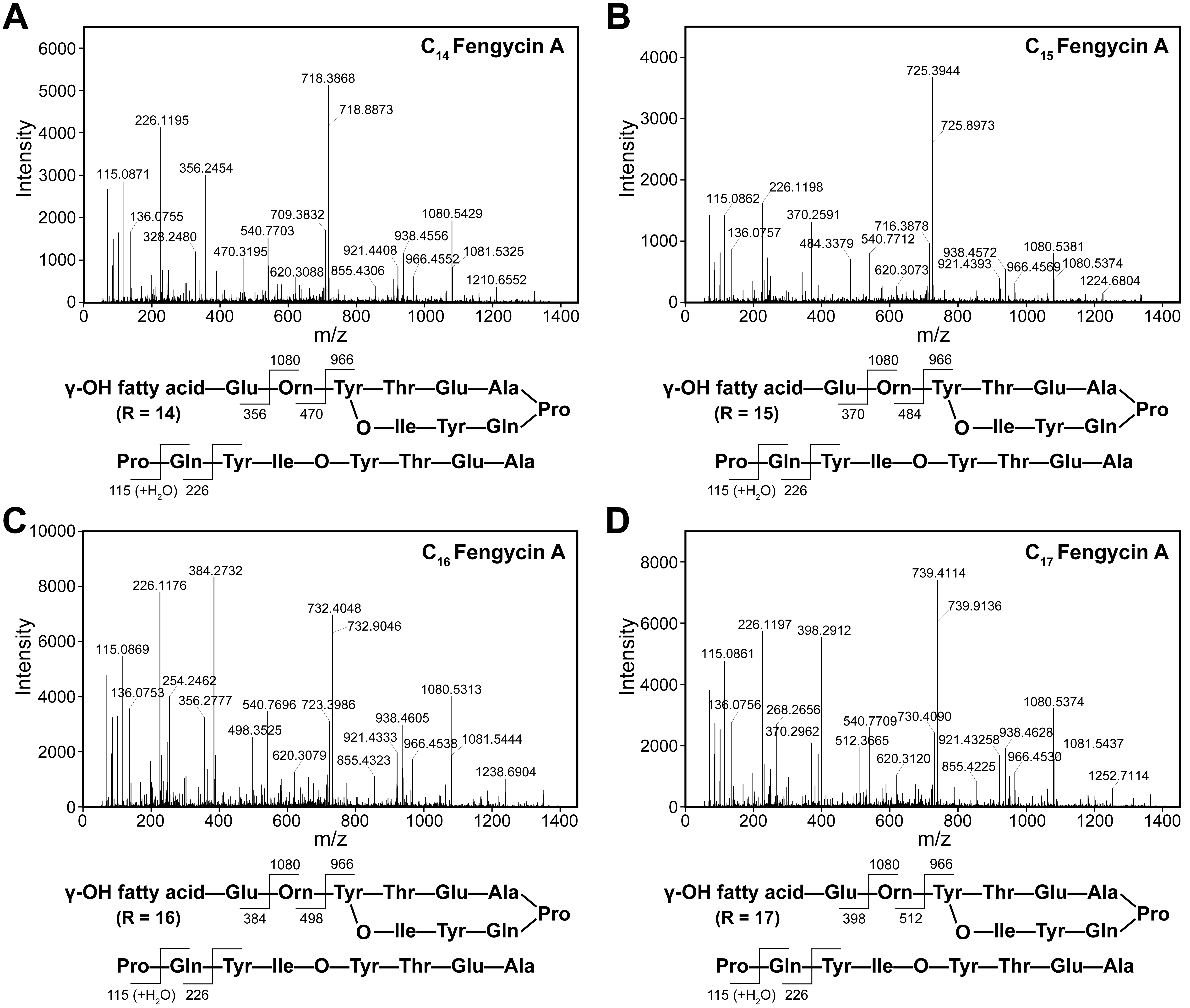
Figure S8. LC-ESI-TOF-MS/MS spectra of [M+2H]^2+^ ions of fengycin precursors. A)** Fengycin A precursor ion at *m/z* 718, containing a C_14_ γ-hydroxy fatty acid chain. **B)** Fengycin A precursor ion at *m/z* 725, containing a C_15_ γ-hydroxy fatty acid chain. **C)** Fengycin A precursor ion at *m/z* 732, containing a C_16_ γ-hydroxy fatty acid chain. **D)** Fengycin A precursor ion at *m/z* 739, containing a C_17_ γ-hydroxy fatty acid chain.

**
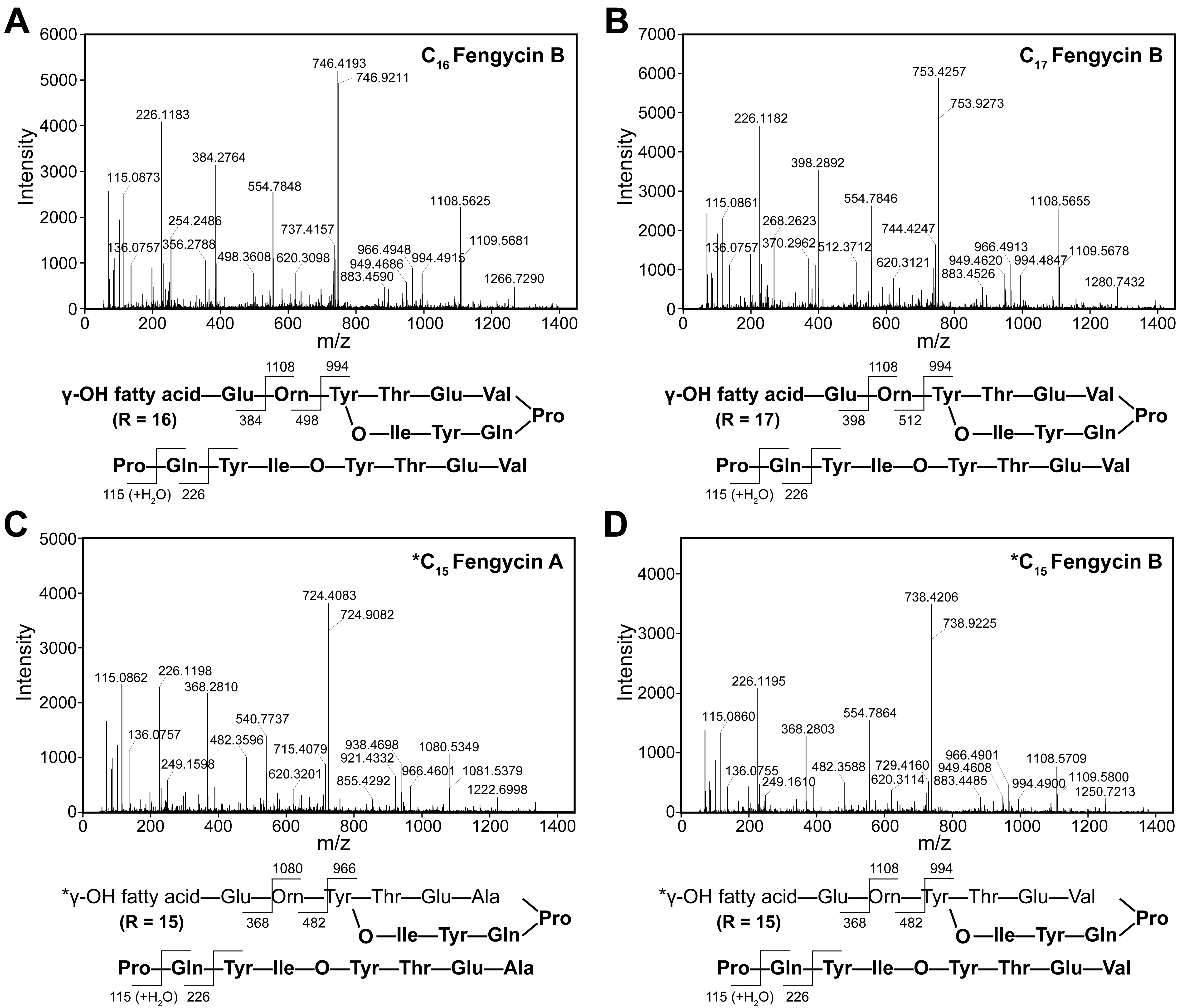
**

**Figure S9. LC-ESI-TOF-MS/MS spectra of [M+2H]^2+^ ions of fengycin precursors. A)** Fengycin B precursor ion at *m/z* 746, containing a C_16_ γ-hydroxy fatty acid chain. **B)** Fengycin B precursor ion at *m/z* 753, containing a C_17_ γ-hydroxy fatty acid chain. **C)** Fengycin A precursor ion at *m/z* 732, containing an unsaturated (*) C_15_ γ-hydroxy fatty acid chain. **D)** Fengycin B precursor ion at *m/z* 739, containing an unsaturated (*) C_15_ γ-hydroxy fatty acid chain.

**
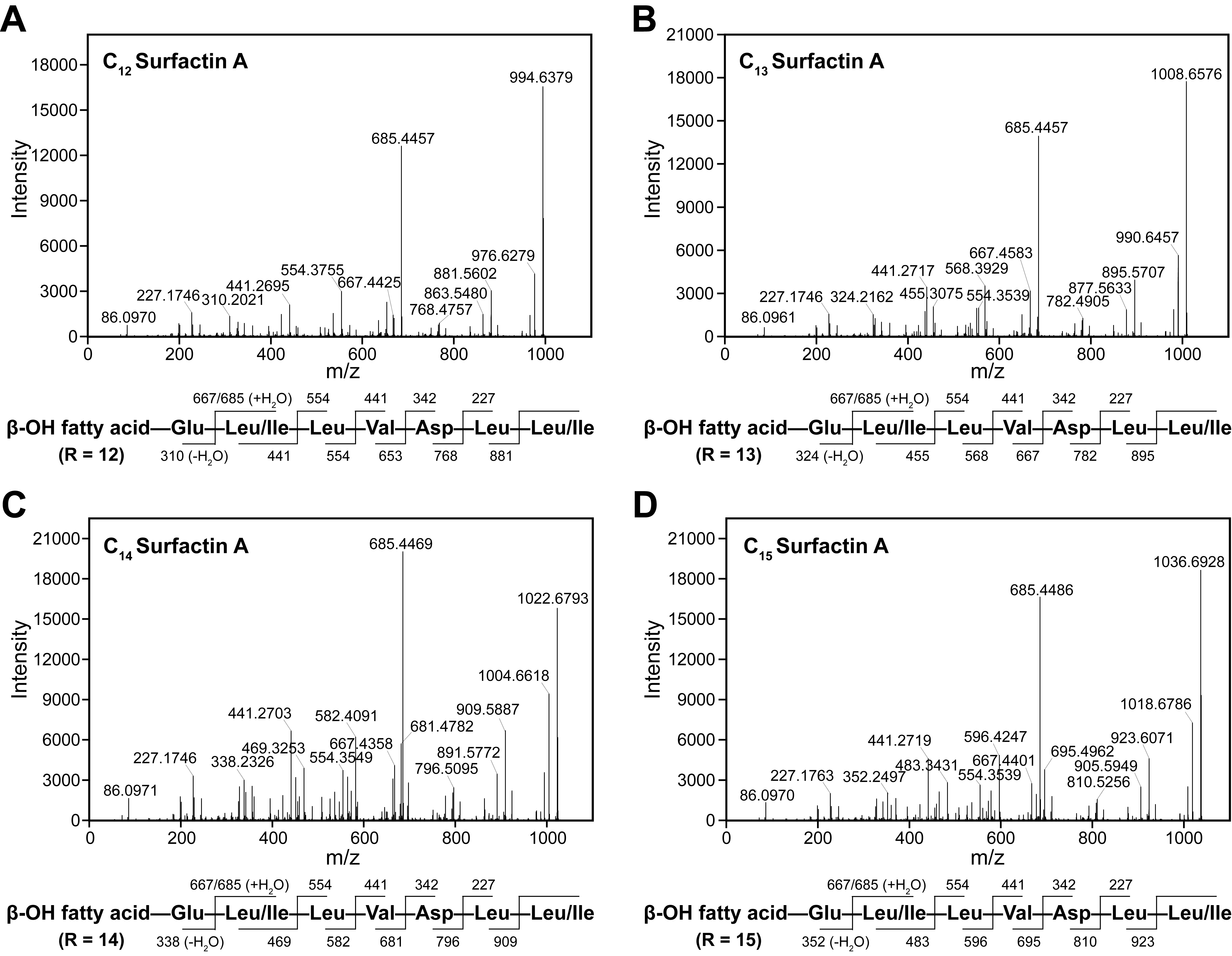
**

**Figure S10. LC-ESI-TOF-MS/MS spectra of [M+H]^+^ ions of surfactin precursors. A)** Surfactin A precursor ion at *m/z* 994, containing a C_12_ β-hydroxy fatty acid chain. **B)** Surfactin A precursor ion at *m/z* 1008, containing a C_13_ β-hydroxy fatty acid chain. **C)** Surfactin A precursor ion at *m/z* 1022, containing a C_14_ β-hydroxy fatty acid chain. **D)** Surfactin A precursor ion at *m/z* 1036, containing a C_15_ β-hydroxy fatty acid chain.


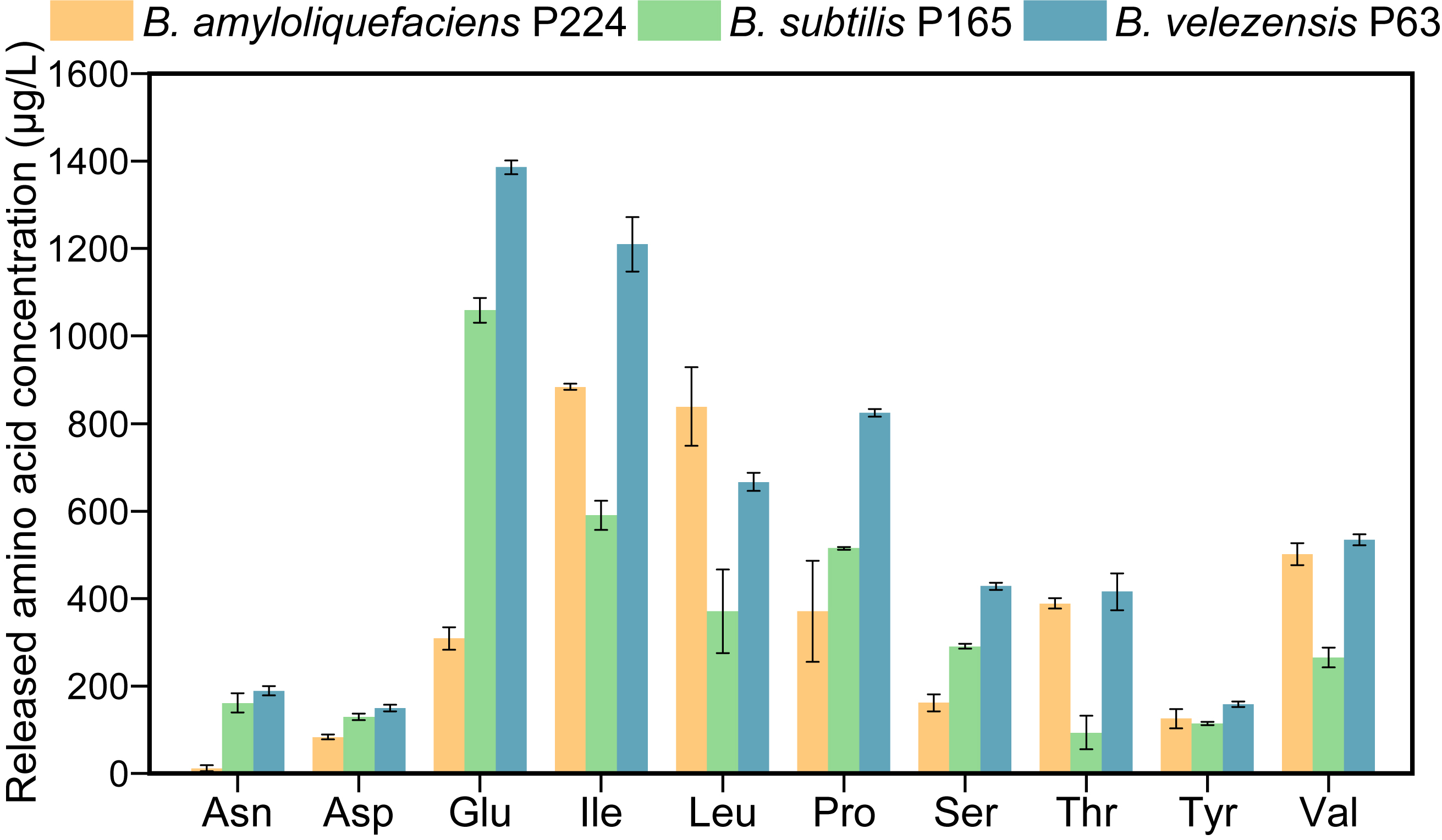


**Figure S11. LC-MS/MS quantification of the eleven amino acids released from *Bacillus* lipopeptides following enzymatic degradation by *S. maltophilia*.** The y-axis zero baseline on the y-axis represents the combined amino acid content from control groups (lipopeptide extract alone + P373 intracellular extract alone). Columns represent the concentration difference in amino acids between treatment groups (lipopeptides mixed with P373 intracellular enzymes) and control groups. The quantified amino acids include: Asn (Asparagine), Asp (Aspartic acid), Glu (Glutamic acid), Ile (Isoleucine), Leu (Leucine), Pro (Proline), Ser (Serine), Thr (Threonine), Tyr (Tyrosine), and Val (Valine).

**
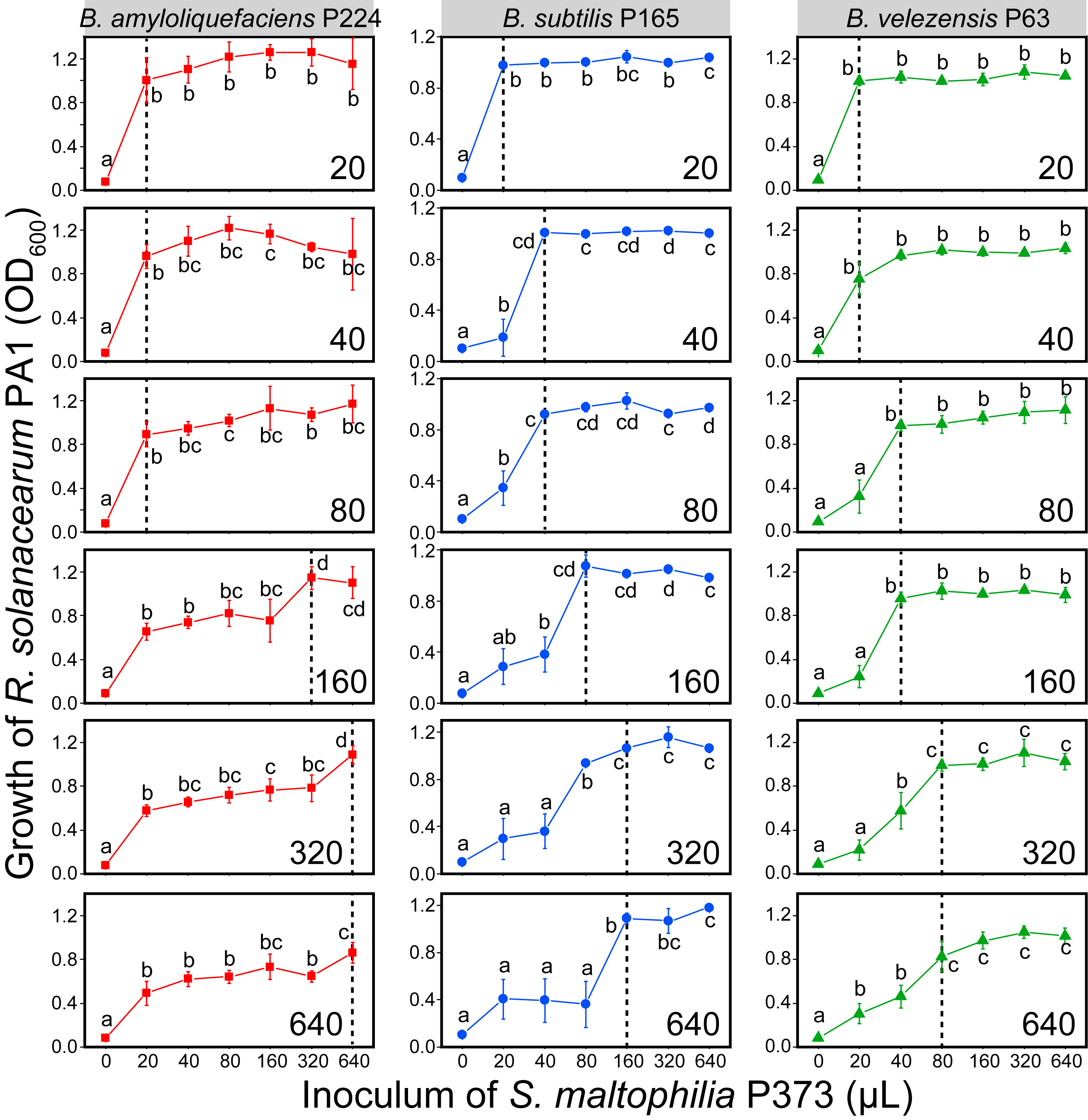
**

**Figure S12. The growth of *R. solanacearum* at 24 h in the presence of varying amounts of *S. maltophilia* and *Bacillus* co-culture supernatants.** Dashed lines represent thresholds beyond which further increases in the inoculum of *S. maltophilia* did not further improve the growth of *R. solanacearum* (one-way ANOVA, *P* < 0.05). The number in the bottom right corner of each plot indicates the amount of *Bacillus* strain inoculum used in the experiment.

**
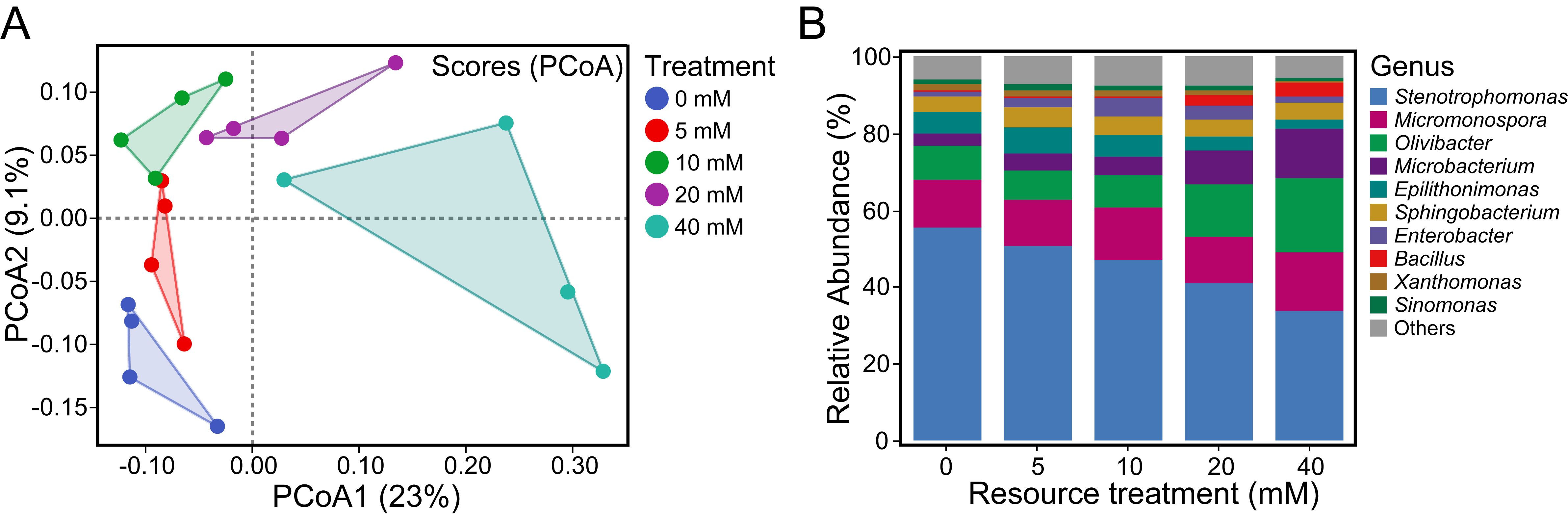
**

**Figure S13. Changes in bacterial community composition in response to carbon resource availability in the soil microcosm experiment.** Principal coordinate analysis (PCoA) based on Bray–Curtis distance (A) and genus-level composition (B) of soil bacterial communities under different carbon resource availability.

**Supplementary Methods**

**Supernatant exposure assays evaluating the direct interactions between *S. maltophilia* and *R. solanacearum***

To exclude the possibility of direct growth promotion on *R. solanacearum* PA1 by *S. maltophilia* P373 in the tripartite interaction patterns, we assessed the growth of PA1 in the presence of P373 culture supernatant. Briefly, 5 μL of overnight pathogen culture (OD_600_ = 0.1) were mixed in a 96-well plate with 20 μL, 50 μL, 100 μL, and 200 μL of P373 cell-free supernatants and the total volume in each well was adjusted to 200 μL with fresh BG medium. Each treatment had four replicates. Uninoculated supernatant controls were included to verify the absence of residual cells. The plates were incubated at 30°C with shaking (150 rpm) for 24 h, after which the growth of pathogen *R. solanacearum* PA1 was quantified by measuring the OD_600_ absorbance of cultures using a SpectraMax M5 microplate reader. The complete absence of detectable growth (OD_600_ < 0.05) in uninoculated control wells confirmed effective cell removal and validated the experimental results. Additionally, the same protocol was used to evaluate the growth of P373 in the presence of the pathogen PA1 cell-free supernatants.

**Toxicity assessments of *Bacillus* culture supernatants post-lipopeptide extraction against *R. solanacearum***

To evaluate the role of lipopeptides in the antimicrobial activity of *Bacillus* metabolites against *R. solanacearum* PA1, we compared the growth of PA1 in the presence of *Bacillus* culture supernatants and lipopeptide-extracted *Bacillus* supernatants (processed by acid precipitation). Briefly, both control and lipopeptide-depleted supernatant preparations were adjusted to pH 7.0 with 6 M KOH and sterilized through 0.22 μm filters. Serial volumes (20 μL, 50 μL, 100 μL, and 200 μL) of each supernatant were then tested for their toxcity against *R. solanacearum* PA1, following the same protocol as above.

**Quantitative LC-MS/MS analysis of amino acids in *Bacillus* lipopeptides following *S. maltophilia* degradation**

The lipopeptide degradation assay was performed using a mixture of 9 mL of lipopeptide-induced *S. maltophilia* P373 cellar extracts and 1000 μL of lipopeptide extracts, as described previously. The control samples consisted of P373 cell extract-only and lipopeptide extract-only groups. All treatments and controls were conducted in triplicate. The quantification of amino acids in lipopeptide deradation products was performed by LC-MS/MS using an HPLC system coupled to a triple quadrupole mass spectrometer (Nexera with LCMS-8050, Shimadzu, Japan) in positive ionization mode. The separation was achieved using a Zorbax Eclipse-AAA column (4.6 mm×150 mm, 3.5 μm; Agilent, USA) maintained at 30 ºC. The mobile phases comprised (A) water containing 0.08% formic acid and 1.2 mM ammonium acetate and (B) methanol, and analytes were resolved in the following elution gradient: 0-1.0 min, 98% A; 1.1-10.0 min, 98% to 70% A; 10.1-11.0 min, 70% to 30% A; 11.1-12.0 min, 30% to 5% A; 12.1-13.5 min, 5% A; and 13.6-16.5 min, 98% A. The flow rate was 0.5 mL min^-1^, and 5 µL sample aliquots were injected using an autosampler maintained at 15 ºC.

Eleven amino acids—asparagine (Asn), aspartic acid (Asp), glutamic acid (Glu), isoleucine (Ile), leucine (Leu), proline (Pro), serine (Ser), threonine (Thr), tyrosine (Tyr), valine (Val)—were quantified as they constitute the cyclic peptide core of the lipopeptides under study. For calibration, a series of standard solutions containing each amino acid at concentrations of 10, 20, 50, 100, 200, 500, 1000, and 2000 μg/L were prepared. The corresponding mass spectrometry peak intensities were acquired for all concentration points. Individual calibration curves were then constructed by plotting the peak intensity against nominal concentrations, enabling quantitative determination of each amino acid in the samples. The released amino acid content was calculated by subtracting the combined background levels from control groups (lipopeptide extract alone and P373 intracellular extract alone) from the measured amino acid content in the treatment group (lipopeptides mixed with P373 intracellular extract).

**References in Supplementary Tables**

1. Bie X, Lu Z, Lu F. Identification of fengycin homologues from *Bacillus subtilis* with ESI-MS/CID. *J Microbiol Meth* 2009; **79**: 272–8. https://doi.org/10.1007/s11356-015-5826-3

2. Chen H, Wang L, Su CX et al. Isolation and characterization of lipopeptide antibiotics produced by *Bacillus subtilis*. *Lett Appl Microbiol* 2008; **47**: 180–6. https://doi.org/10.1111/j.1472-765X.2008.02412.x

3. Daas MS, Acedo JZ, Rosana ARR et al. *Bacillus amyloliquefaciens* ssp. *plantarum* F11 isolated from Algerian salty lake as a source of biosurfactants and bioactive lipopeptides. *FEMS Microbiol Lett* 2018; **1**: 365. https://doi.org/10.1093/femsle/fnx248

4. Gong M, Wang JD, Zhang J et al. Study of the antifungal ability of *Bacillus subtilis* strain PY-1 in vitro and identification of its antifungal substance (Iturin A). *Acta Bioch Bioph Sin* 2006; **38**: 233–40. https://doi.org/10.1111/j.1745-7270.2006.00157.x

5. Ma Y, Kong Q, Qin C, Chen Y et al. Identification of lipopeptides in *Bacillus megaterium* by two-step ultrafiltration and LC-ESI-MS/MS. *AMB Expr* 2016; **6**: 79. https://doi.org/10.1186/s13568-016-0252-6

6. Pecci Y, Rivardo F, Martinotti MG, et al. LC/ESI‐MS/MS characterisation of lipopeptide biosurfactants produced by the *Bacillus licheniformis* V9T14 strain. *J Mass Spectrom* 2010; **45**: 772–8. https://doi.org/10.1002/jms.1767

7. Su Z, Chen X, Liu X et al. Genome mining and UHPLC–QTOF–MS/MS to identify the potential antimicrobial compounds and determine the specificity of biosynthetic gene clusters in *Bacillus subtilis* NCD-2. *BMC Genomics* 2020; **21**: 767. https://doi.org/10.1186/s12864-020-07160-2

8. Yang H, Li X, Li X et al. Identification of lipopeptide isoforms by MALDI-TOF-MS/MS based on the simultaneous purification of iturin, fengycin, and surfactin by RP-HPLC. *Anal Bioanal Chem* 2015; **407**: 2529–42. https://doi.org/10.1007/s00216-015-8486-8
